# Structural and evolutionary insights into DAF-12 interactions with transcriptional coactivators in parasitic nematodes

**DOI:** 10.1371/journal.ppat.1014073

**Published:** 2026-07-20

**Authors:** Manon Mallet, Yéléna Martin, João E. Carvalho, Eva Guchen, Remy Betous, Cherine Bechara, Anne Lespine, Michael Schubert, William Bourguet, Albane le Maire

**Affiliations:** 1 Centre de Biologie Structurale (CBS), University of Montpellier, INSERM, CNRS, Montpellier, France; 2 Laboratoire de Biologie du Développement de Villefranche-sur-Mer, Institut de la Mer de Villefranche, Sorbonne Université, CNRS, Villefranche-sur-Mer, France; 3 INTHERES, Université de Toulouse, INRAE, ENVT, Toulouse, France; 4 IGF, Université de Montpellier, CNRS, INSERM, Montpellier, France; University of Wisconsin-Madison, UNITED STATES OF AMERICA

## Abstract

Parasitic nematodes infect billions of humans and livestock worldwide, causing major health and economic burdens, while the spread of anthelmintic resistance threatens current control strategies. A critical step in parasite infection is the resumption of development of infective third-stage larvae (iL3) upon host entry, a process controlled by the nuclear receptor DAF-12. Activation of DAF-12 by dafachronic acids promotes developmental progression and reproductive maturation, making this receptor an attractive therapeutic target. However, the molecular mechanisms governing DAF-12 activation, particularly transcriptional coactivator recruitment, remain poorly understood. Here, we combined biophysical, cellular, structural, and bioinformatic approaches to investigate coactivator recognition by DAF-12 from the parasitic nematodes *Brugia malayi* and *Haemonchus contortus*. Crystal structures of ligand-bound DAF-12 ligand-binding domains in complex with coactivator-derived peptides reveal conserved features of ligand-dependent coactivator recruitment shared with mammalian nuclear receptors. In addition, we uncover previously unrecognized interaction features, including motif-specific contacts that extend beyond the canonical LXXLL binding mode of coactivators and distinct patterns of DAF-12 conservation across nematode clades. Structure-guided analyses redefine the interaction motif of the only described parasite-specific coactivator DIP-1 and suggest novel candidate motifs for DAF-12-interacting proteins. Together, these findings establish the structural basis of coactivator binding to nematode DAF-12 and provide mechanistic insight into the transcriptional regulation underlying parasite development. These results expand current understanding of nuclear receptor signaling in parasitic nematodes and provide a framework for the future design of strategies aimed at disrupting DAF-12 activation as a potential antiparasitic approach.

## Author summary

Parasitic nematodes infect billions of people and livestock worldwide, causing major health and economic burdens, while increasing resistance threatens current treatments. These parasites rely on a developmental switch that allows infectious larvae to resume growth inside their host, a process controlled by the nuclear receptor DAF-12. Blocking this pathway could prevent parasites from establishing infection. However, the molecular mechanisms regulating DAF-12 activation remain poorly understood. Here, we investigate how DAF-12 from two parasitic nematodes, *Brugia malayi* and *Haemonchus contortus*, interacts with transcriptional coactivators that enable gene activation, using a combination of biophysical, cellular, structural, and bioinformatic approaches. We identified conserved features of ligand-dependent coactivator recruitment shared with mammalian nuclear receptors as well as nematode-specific interaction mechanisms that vary across evolutionary clades. These findings provide new insights into the structural basis of coactivator binding to DAF-12 and advance our understanding of a key pathway controlling parasitic nematode development.

## Introduction

A vast number of nematode parasites are known to infect humans, causing an important public health problem. Parasitic nematodes infect nearly three billion people worldwide, and are responsible for several neglected tropical diseases (NTDs) prioritized by the World Health Organization [1], among them river blindness, cutaneous and lymphatic filariasis caused by filarial worms such as *Onchocerca volvulus* or *Brugia malayi*. Beyond their impact on human health, parasitic nematodes are also major pathogens in domesticated animals, as seen with *Dirofilaria immitis* and *Dirofilaria repens*, which cause zoonotic dirofilariasis. Additionally, parasitic nematodes severely alter animal welfare and food production, leading to substantial economic loss for the livestock industry. The global veterinary market for anthelmintic pharmaceuticals exceeds $3.5 billion annually, further exacerbated by mortality and productivity loss. Among the most problematic species, the gastrointestinal nematode *Haemonchus contortus*, infecting small ruminants, is the most prevalent species due to its high pathogenicity, prolificacy, and genetic variability. These traits increase disease burden and accelerate the development of resistance to multiple anthelmintic treatments, posing a critical threat to sustainable livestock management [2].

Parasitic nematodes belong to distinct evolutionary clades, with markedly different and complex life cycles involving multiple developmental stages and host interactions. For instance, *B. malayi* belongs to the clade III superfamily *Filarioideae* and exhibits a complex life cycle involving multiple developmental stages in both an arthropod vector and the definitive mammalian host [3,4]. *B. malayi* infects the human lymphatic system, where adult females release microfilariae (L1 larvae) into the bloodstream. They are ingested by mosquitoes, in which the microfilariae develop into infective L3 larvae (iL3), and re-enter the human host upon a mosquito bite, maturing into adults in the lymphatic system. By contrast, *H. contortus*, a soil-borne nematode belonging to the clade V superfamily *Trichostrongyloidea*, follows a free-living phase before ending its life cycle within a ruminant host [5,6]. Adults localize in the abomasum, where they shed eggs in the feces. These eggs hatch into L1 larvae and develop into iL3 larvae, which mature into adults after ingestion by a new host. Despite these differences in life cycles, parasitic nematodes share common features: (i) they enter their mammalian host as developmentally arrested iL3 larvae, which resume development upon infection, and (ii) subsequently undergo molts to adapt to the host environment and mature into reproductive adults. The iL3 stage is functionally analogous to the dauer larval stage of the free-living nematode *Caenorhabditis elegans*, characterized by larval developmental arrest and resistance to harsh environments.

In *C. elegans*, DAF-12 plays a key role in regulating entry into and exit from the quiescent dauer stage [7]. This process is controlled by dafachronic acids, which act as molecular signals to trigger developmental transitions. Under unfavorable conditions, such as food deprivation, *C. elegans* larvae stop producing dafachronic acids and enter into quiescence. When environmental conditions improve, dafachronic acid synthesis resumes, activating DAF-12, which in turn induces expression of reproductive development genes and facilitates dauer exit. Consequently, inhibiting *C. elegans* DAF-12 activation prevents exit from quiescence and stops reproductive development [8]. Similarly, a large body of evidence supports the critical role of DAF-12 in regulating the infectious processes of various parasitic nematodes [9–15], by controlling the transition from the L3 to the adult stage. *B. malayi* and *D. immitis* DAF-12 bind the natural Δ4-dafachronic acid (Δ4-DA) with significantly higher affinity than their counterparts in non-filarial nematodes, such as *H. contortus* and *C. elegans* [16]. This ligand triggers the exit from the iL3 to the adult stage upon infection of the mammalian host. Thus, inhibiting DAF-12 in these nematodes prevents iL3 larvae from resuming development, thereby blocking the parasites from settling in their host and interrupting growth and reproduction of the nematodes. Targeting DAF-12 thus represents a valid option for the development of novel therapeutic strategies to combat nematode parasites. Importantly, the potential therapeutic benefit of targeting DAF-12 has already been validated in *Strongyloides stercoralis* [17].

DAF-12 is a ligand-regulated transcription factor belonging to the nuclear receptor superfamily, whose activity is tuned by transcriptional coregulators (coactivators and corepressors). While many coregulators have been identified and found to be associated with numerous diseases in humans [18], only a few have been described in nematodes. A parasite-specific coactivator, called DIP-1, required for DAF-12 ligand-dependent transcriptional activity has been identified in *S. stercoralis* [13], whereas DIN-1, a corepressor that binds unliganded DAF-12, to regulate lipid metabolism, larval stage-specific programs, diapause, and longevity has been found in *C. elegans* [19]. The hallmark of nuclear receptors is the presence of a conserved DNA binding domain (DBD) which binds to specific promoters of target genes and a ligand binding domain (LBD) that binds small ligands. Ligand-activated nuclear receptors undergo conformational changes, primarily involving the reorientation of the LBD C-terminal activation helix H12. This conformational rearrangement promotes the formation of a conserved interaction surface, called activation function 2 (AF-2), that specifically recognizes a conserved structural LXXLL motif (X stands for any amino acid) present in most transcriptional coactivators [20–22]. Recruitment of these coactivators to the AF-2 surface facilitates the assembly of transcriptional regulatory complexes and ultimately modulates the expression of target genes.

No antagonists that can prevent coactivator recruitment to DAF-12 and subsequently its activation have been identified to date. In particular, the development of antagonists based on dafachronic acids, that are the ligands of DAF-12 in different nematodes [15–17] poses substantial practical challenges. The structural complexity of these sterol-derived molecules renders their chemical synthesis and diversification technically demanding and economically costly, thereby limiting their utility as starting points for drug development efforts. Additionally, structural insights on DAF-12 proteins remain limited to LBD characterizations from two parasitic nematode species [14,23], and the repertoire of nematode coactivators is largely undefined. This incomplete understanding of the molecular mechanisms governing DAF-12 activation and regulation explains why progress on novel therapeutic strategies targeting this receptor has been limited.

To better elucidate the mechanism of transcriptional coactivator recruitment by nematode DAF-12, we combined biophysical, cellular, and structural analyses of *B. malayi* and *H. contortus* DAF-12 LBDs together with a comprehensive bioinformatic analysis of DAF-12 sequences across nematodes. We first identified conserved features of ligand-dependent coactivator recruitment shared with mammalian nuclear receptors, which were validated by the determination of the crystal structures of *B. malayi* and *H. contortus* DAF-12 LBDs in complex with mammalian coactivator-derived peptides. Moreover, we uncovered DAF-12-specific interactions with coactivators, displaying varying degrees of conservation across nematode clades. In addition to redefining the interaction motif of DIP-1, we propose novel motifs of coactivators that may interact with DAF-12, providing a framework for the identification of transcriptional coactivators in nematodes. Together, our findings, in complement to the studies on *Ancylostoma ceylanicum* and *S. stercolaris* DAF-12 [14,23], establish the structural basis of coactivator binding to DAF-12, advancing our understanding of the molecular mechanisms of action of this key regulator of nematode development.

## Results

### *B. malayi* and *H. contortus* DAF-12 LBD purification and ligand binding

**Fig 1:**
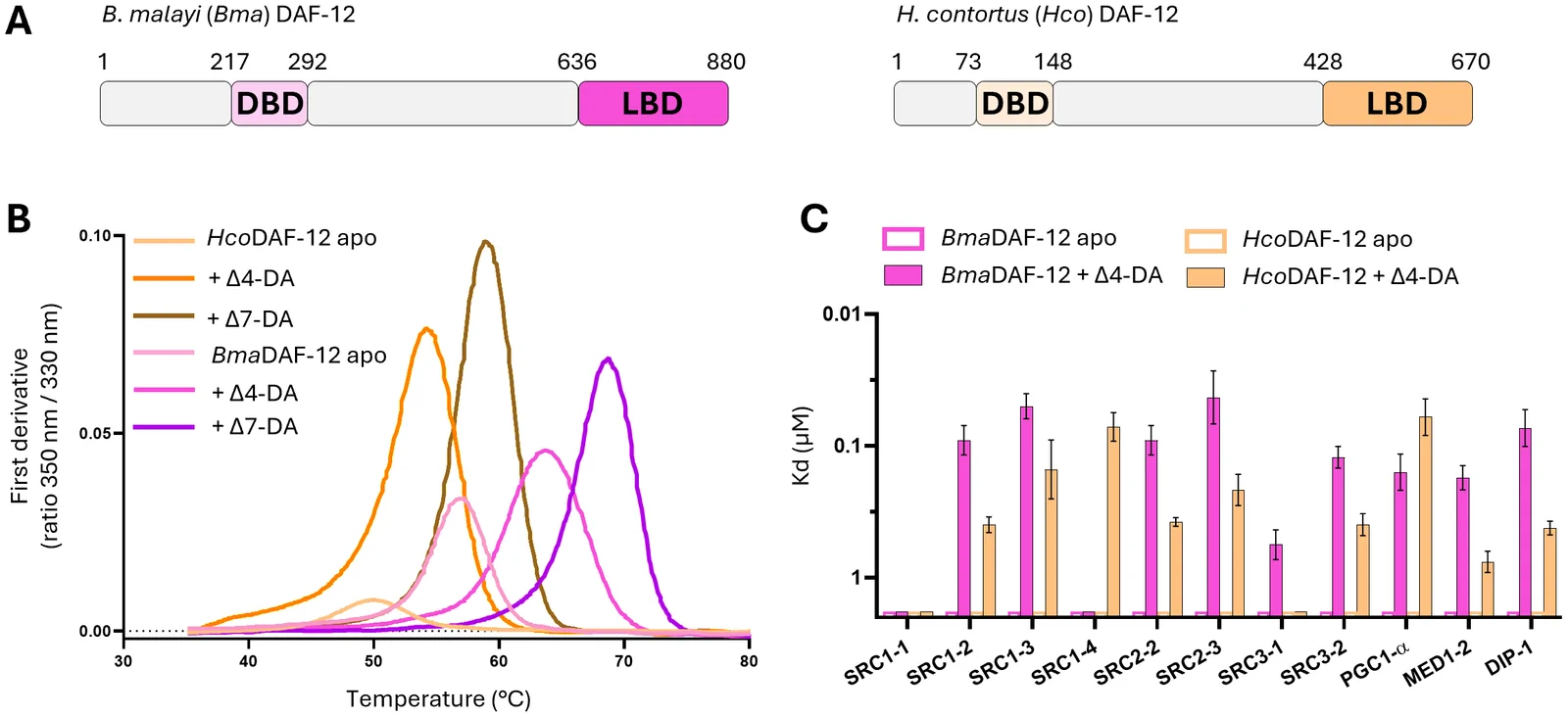
Ligand and coactivator binding properties of *Brugia malayi* (*Bma*) and *Haemonchus contortus* (*Hco*) DAF-12 ligand binding domains. **A.** Schematic representation of *Bma*DAF-12 and *Hco*DAF-12 showing the DNA binding domain (DBD) and ligand binding domain (LBD) limits. **B.** Nano-DSF analysis of ligand binding to *Bma*DAF-12 and *Hco*DAF-12 LBDs. ∆4- and ∆7-dafachronic acids (∆4-DA and ∆7-DA) or DMSO (apo conditions) were added in one molar equivalent to the proteins. The first derivative of the ratio between intrinsic fluorescence at 350 nm and 300 nm is plotted against the temperature. The experiments were repeated three times independently. **C.** Affinities of *Bma*DAF-12 and *Hco*DAF-12 LBDs for different peptides derived from coactivators, in the absence and in the presence of ∆4-DA. Dissociation constants (Kd in µM) were derived from fluorescence anisotropy experiments.

We structurally and functionally characterized the LBD of DAF-12 from a filarial parasite, *B. malayi* (*Bma*) and a non-filarial parasite, *H. contortus* (*Hco*), which share 55% sequence identity [16] (Fig 1A). Both proteins were expressed in a bacterial system and purified to high purity (>99% for *Bma*DAF-12 and >95% for *Hco*DAF-12) (S1A and S1B Fig). Initial attempts to express the *Hco*DAF-12 LBD yielded a low amount of soluble protein. To improve solubility, we introduced four cysteine-to-serine mutations (C538S, C592S, C610S, C646S), following an approach previously used for *A. ceylanicum (Ace*) DAF-12 [23] (S1C Fig). Due to their location in the LBD out of the ligand and coregulator binding sites, these mutations are not expected to affect ligand or coregulator binding. The mutated variant (hereafter *Hco*DAF-12) was markedly more soluble than the wild-type receptor, yielding over 5 mg of pure protein per liter of culture and was thus used for all the following biophysical and structural analyses. The structural integrity of the purified proteins was verified by circular dichroism (CD) and dynamic light scattering (DLS). CD spectra indicated that they have a high 𝛼-helical content (60.1% for *Bma*DAF-12 and 74.1% for *Hco*DAF-12) (S1D Fig), consistent with the folded conformation of a nuclear receptor LBD. DLS measurements showed a hydrodynamic radius of 2.4 and 2.6 nm for *Bma*DAF-12 and *Hco*DAF-12, respectively (S1E and S1F Fig), consistent with a monomeric form of the DAF-12 LBDs in solution.

To demonstrate ligand binding to purified *Bma*DAF-12 and *Hco*DAF-12 LBDs, we analyzed their thermal stability, without and with one or three molar equivalents of Δ4-DA and (25*S*)-Δ7-dafachronic acid (Δ7-DA), using nano-differential scanning fluorimetry (nano-DSF) (Figs 1B, S2A and S2B and S1 Table). The proteins were incubated with the ligands and subjected to a thermal gradient, and the intrinsic tryptophan and tyrosine fluorescence was monitored. Both dafachronic acids significantly stabilized the *Bma*DAF-12 and *Hco*DAF-12 LBDs, even at a 1:1 ligand-to-protein ratio, in agreement with their high potency to active *Bma*DAF-12 and *Hco*DAF-12 [16]. The ligand-induced thermal stabilization of *Hco*DAF-12 is similar to the one measured with the wild-type protein with a ΔTm equal to 3.8 and 8.4 °C (mutant) versus 5.4 and 10°C (wild-type) for Δ4-DA and Δ7-DA, respectively, validating that these mutations do not interfere with ligand binding (S2B Fig) Finally, native mass spectrometry (nMS) analysis of *Bma*DAF-12 incubated with two and a half molar excess of Δ4-DA confirmed complex formation and showed a 1:1 stoichiometry, as evidenced by the appearance of a new distribution corresponding to the expected mass of the protein-ligand complex (29170 Da) (S2C and S2D Fig). Thus, our results confirmed the physical binding of dafachronic acids to *B. malayi* and *H. contortus* DAF-12, in agreement with their functional ligand-dependent activation [16].

### *B. malayi* and *H. contortus* DAF-12 recruit coactivators in a ligand-dependent manner

To analyze the ability of *Bma*DAF-12 and *Hco*DAF-12 LBDs to bind transcriptional coactivators in a ligand-dependent manner, we screened a panel of eleven peptides derived from coactivators using steady-state fluorescence anisotropy experiments. Since transcriptional coregulators from *B. malayi* and *H. contortus* remain uncharacterized, this panel included ten LXXLL-containing peptides derived from mammalian coactivators and one FXXLL-containing peptide derived from *S. stercoralis (Sst)* DIP-1 [13], the only coactivator described to date in nematodes (Figs 1C and S3 and Table 1).

**Table 1:** Coactivator binding to *Brugia malayi* (*Bma*) and *Haemonchus contortus* (*Hco*) DAF-12 ligand binding domains, in the presence of ∆4-DA.

		*Bma*DAF-12 + ∆4-DA	*Hco*DAF-12 + ∆4-DA
Peptide	Sequence	Kd (nM)	Kd (nM)
**hPGC1α-1** (E140-A152)	φ-EPS**LLK**K**LL**LAPA	158 [115; 217]	60 [44; 83]
**hSRC2–3** (Q732-D756)	φ-QEPVSPKKKENA**LLR**Y**LL**DKDDTKD	43 [27; 68]	215 [164; 283]
**hSRC2–2** (K682-S695)	φ-K**H**K**ILH**R**LL**QDSSS	91 [70; 117]	377 [350; 406]
**hSRC1–2** (R686-S698)	φ-R**H**K**ILH**R**LL**QEGS	94 [79; 112]	396 [346; 453]
**hSRC3–2** (T670-G692)	φ-TSNMHGSLLQEK**H**R**ILH**K**LL**QNG	122 [101; 147]	395 [326; 478]
**hSRC1–3** (E742-L760)	φ-ESKD**H**Q**LLR**Y**LL**DKDLKDL	50 [40; 62]	151 [90; 252]
**hMED1–2** (N641-N652)	φ-N**H**P**ML**MN**LL**KDN	174 [141; 215]	757 [628; 911]
**sstDIP-1** (V49-I65)	φ-VIEGSEK**F**P**TL**LA**AI**RK	73 [53; 101]	419 [372; 472]
**hSRC1–4** (Q1430-E1441)	φ-QQKS**LL**QQ**LL**TE	100 [74; 133]	72 [56; 92]
**hSRC3–1** (A606-S629)	φ-AENQRGPLESKG**H**KK**L**LQ**LL**TCSS	562 [433; 729]	> 2 µM
**hSRC1–1** (K625-L645)	φ-KYSQTSHK**L**VQ**LL**TTTAEQQL	> 10 µM	> 10 µM
(K632L)	φ-KYSQTSH**LL**VQ**LL**TTTAEQQL	984 [742; 1306]	2460 [1944; 3112]
(V634K)	φ-KYSQTSHK**LK**Q**LL**TTTAEQQL	> 10 µM	> 10 µM
(S630H/V634H)	φ-KYSQT**H**HK**LH**Q**LL**TTTAEQQL	814 [675; 984]	3420 [2393; 1890]
(S630H/K632L/V634K)	φ-KYSQT**H**H**LLK**Q**LL**TTTAEQQL	151 [100; 267]	771 [616; 966]

Dissociation constants (Kd in µM) and associated confidence range were derived from fluorescence anisotropy experiments, performed in triplicate. Colored residues in the different peptides correspond to amino acids discussed in the manuscript, and φ corresponds to the position of the fluorescein.

As expected, no coactivator binding was measured in the absence of dafachronic acids, while in the presence of Δ4-DA, several coactivator-derived peptides were efficiently recruited by *Bma*DAF-12 and *Hco*DAF-12 LBDs. The *Hco*DAF-12 LBD displayed the highest affinities for PGC1⍺-1 and SRC1–4 peptides, as also observed for DAF-12 from *A. ceylanicum*, *Ancylostoma caninum*, *Necator americanus*, and *S. stercolaris* [23]. The *Bma*DAF-12 LBD showed higher affinities for coactivator-derived peptides in general and distinct preferences with affinities ranging from 43 to 174 nM for most of the peptides derived from coactivators belonging to different families. These observations suggest species-specific coactivator preferences among DAF-12 orthologs and suggest that residues flanking the LXXLL motif contribute to both binding affinity and receptor specificity. In addition, both DAF-12 LBDs interacted weakly with the motifs 1 of SRC1 and SCR3. Both *Bma*DAF-12 and *Hco*DAF-12 LBDs also interacted with the *Sst*DIP-1 FXXLL-containing peptide in the presence of Δ4-DA with Kd values of 73 nM and 419 nM, respectively. As expected, ∆7-DA also induced *Sst*DIP-1 binding with comparable affinities (S3C Fig), confirming the agonistic activity of both dafachronic acids [16].

**Fig 2:**
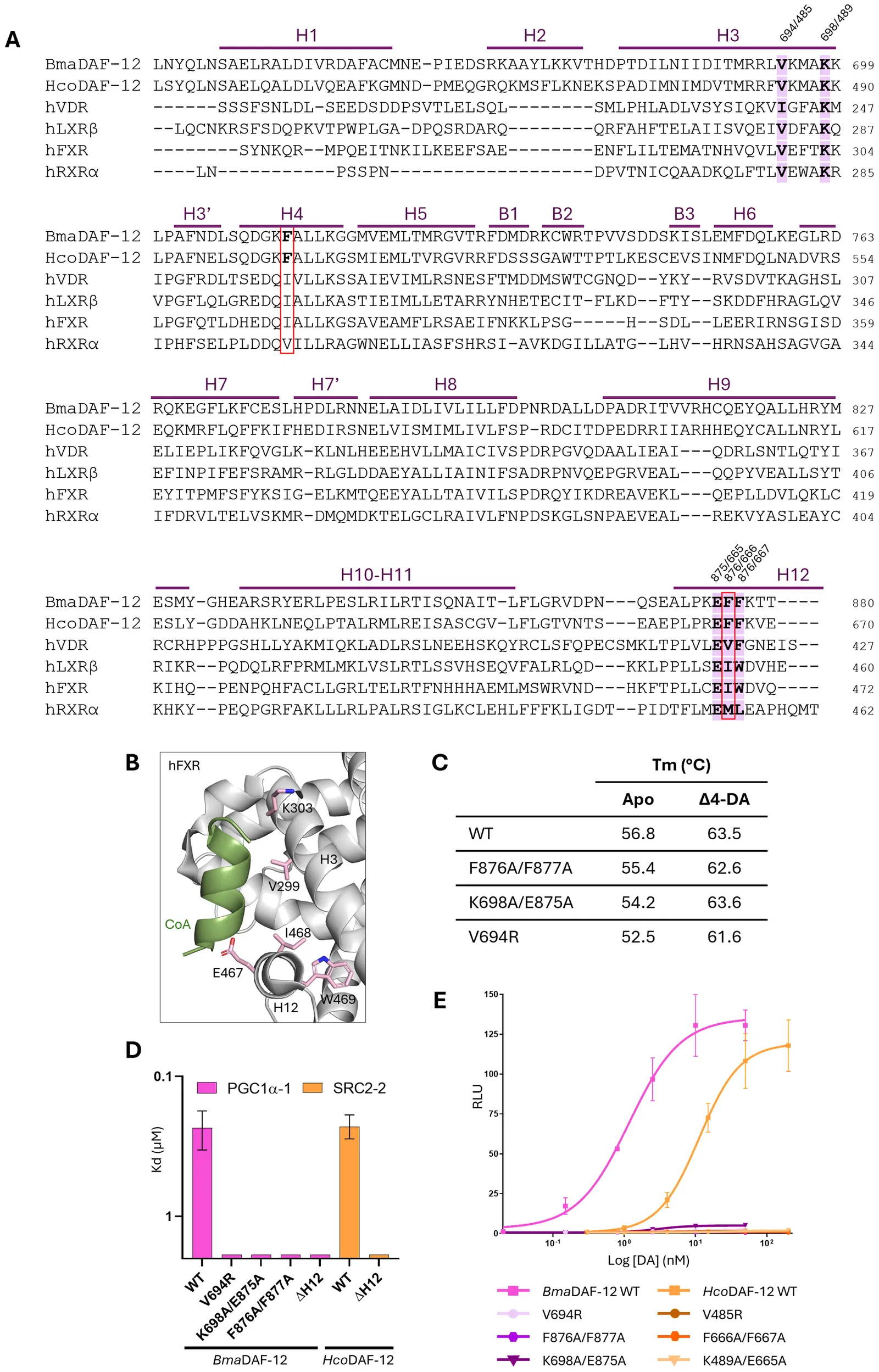
Validation of a conserved coactivator binding mode between *Brugia malayi* (*Bma*) and *Haemonchus contortus* (*Hco*) DAF-12 and mammalian nuclear receptors. **A.** Sequence alignment of the LBDs of the *Brugia malayi* (*Bma*) DAF-12, the *Haemonchus contortus* (*Hco*) DAF-12, the closest human nuclear receptors hVDR, hLXRβ and hFXR and the more distant nuclear receptor, RXRα. Residues involved in coactivator recruitment are highlighted in purple. The two phenylalanines conserved in DAF-12 but not present in human nuclear receptors are framed in red. Numbering of mutated residues from *Bma*DAF-12 and *Hco*DAF-12 are indicated above the alignment, as well as secondary structures elements. **B.** Close-up view of the *h*FXR LBD-SRC1 coactivator peptide (CoA) interface from the crystal structure (PDB 3BEJ) [26]. Residues involved in binding are shown as sticks and labeled. **C.** Inflection temperatures deduced from nano-DSF experiments of wild-type (WT) and mutant *Bma*DAF-12 LBDs in their apo state and in the presence of three molar excess of ∆4-DA. **D.** Affinities of WT and mutant *Bma*DAF-12 for the PGC1α-1 peptide (pink), and of *Hco*DAF-12 and *Hco*DAF-12∆H12 for the SRC2-2 peptide (orange). Dissociation constants (Kd in µM) were derived from fluorescence anisotropy experiments. **E.** Dose-response curves for Δ4-DA on the activation of WT and mutant *Bma*DAF-12 (pink) and for ∆7-DA on the activation of WT and mutant *Hco*DAF-12 (orange). NIH3T3 cells were co-transfected with Gal4-DAF-12_LBD and the luciferase gene reporter construct. Transfected cells were incubated with increasing concentrations of dafachronic acids (DA; Δ4-DA for *Bma*DAF-12 and Δ7-DA for *Hco*DAF-12). Transactivation activity was assessed by measuring luciferase activity normalized first to Renilla luciferase activity to control for transfection efficiency, and then to the firefly/Renilla luciferase ratio obtained from cells transfected with a control vector expressing only Gal4-DBD and treated under the same conditions. Relative DAF-12 activities are expressed as relative light units (RLU). Data representing normalized luciferase activities are plotted with nonlinear regression fit using sigmoidal dose response with variable slope (Prism 6.0, Graph Pad Software, Inc.). Values are means from two independent experiments performed in technical triplicates.

These observations also suggest a mode of coactivator binding conserved between mammalian and nematode nuclear receptors. To further substantiate this hypothesis, we performed mutagenesis of DAF-12 conserved residues, which are known to play a critical role in coactivator recruitment by mammalian nuclear receptors [24], in particular the charge clamp K303/E467 in hFXR [25] (Fig 2A and 2B). After checking their conserved ligand-binding ability compared to wild-type *Bma*DAF-12 (Fig 2C), we performed fluorescence anisotropy assays with *Bma*DAF-12 mutants to assess their binding to the PGC1α-1 peptide (Figs 2D and S3D). Substitution of V694 (V299 in hFXR) within the hydrophobic groove of DAF-12 that interacts with the leucine residues of coactivator peptides with a charged residue (V694R) abolished peptide interaction. Similarly, alanine substitution of the charged clamp residues K698A and E875A (K303 and E467 in hFXR) involved in hydrogen bonds with coactivator peptides eliminated coactivator binding. We next tested whether the active positioning of helix H12 is also stabilized by hydrophobic interactions as observed with I468 and W469 in hFXR. Alanine substitution of the corresponding residues, F876 and F877, prevented the stabilized position of helix H12 and abolished PGC1α-1 binding. Importantly, the residues involved in coactivator recognition are highly conserved between *Bma*DAF-12 and *Hco*DAF-12. Therefore, we would expect the corresponding mutations in *Hco*DAF-12 to produce effects similar to those observed for the purified *Bma*DAF-12 mutants. To further assess the role of H12, we generated deletion constructs lacking this helix (*Bma*DAF-12ΔH12 and *Hco*DAF-12ΔH12). These variants failed to recruit coactivator peptides in the presence of Δ4-DA (Figs 2D, S3D and S3E). Consistent with these findings, luciferase-based transactivation assays conducted in NIH3T3 cells showed that none of the *Bma*DAF-12 mutants exhibited transcriptional activity in the presence of Δ4-DA, in contrast to robust luciferase expression by wild-type *Bma*DAF-12 (EC50 = 1.2 nM) (Fig 2E). Equivalent mutations in *Hco*DAF-12 (V485R, K489A/E665A, F666A/F667A) (Fig 2A) similarly abrogated receptor activation in response to Δ7-DA, contrary to the strong luciferase expression by wild-type *Hco*DAF-12 (EC50 = 11.3 nM) (Fig 2E). Together, these results underscore the critical role of helix H12 in mediating coactivator recruitment and confirm that DAF-12 orthologs in parasitic nematodes utilize a conserved activation mechanism closely resembling that of mammalian nuclear receptors.

### Crystal structures of *B. malayi* and *H. contortus* DAF-12 LBDs in complex with coactivator peptides

**Fig 3:**
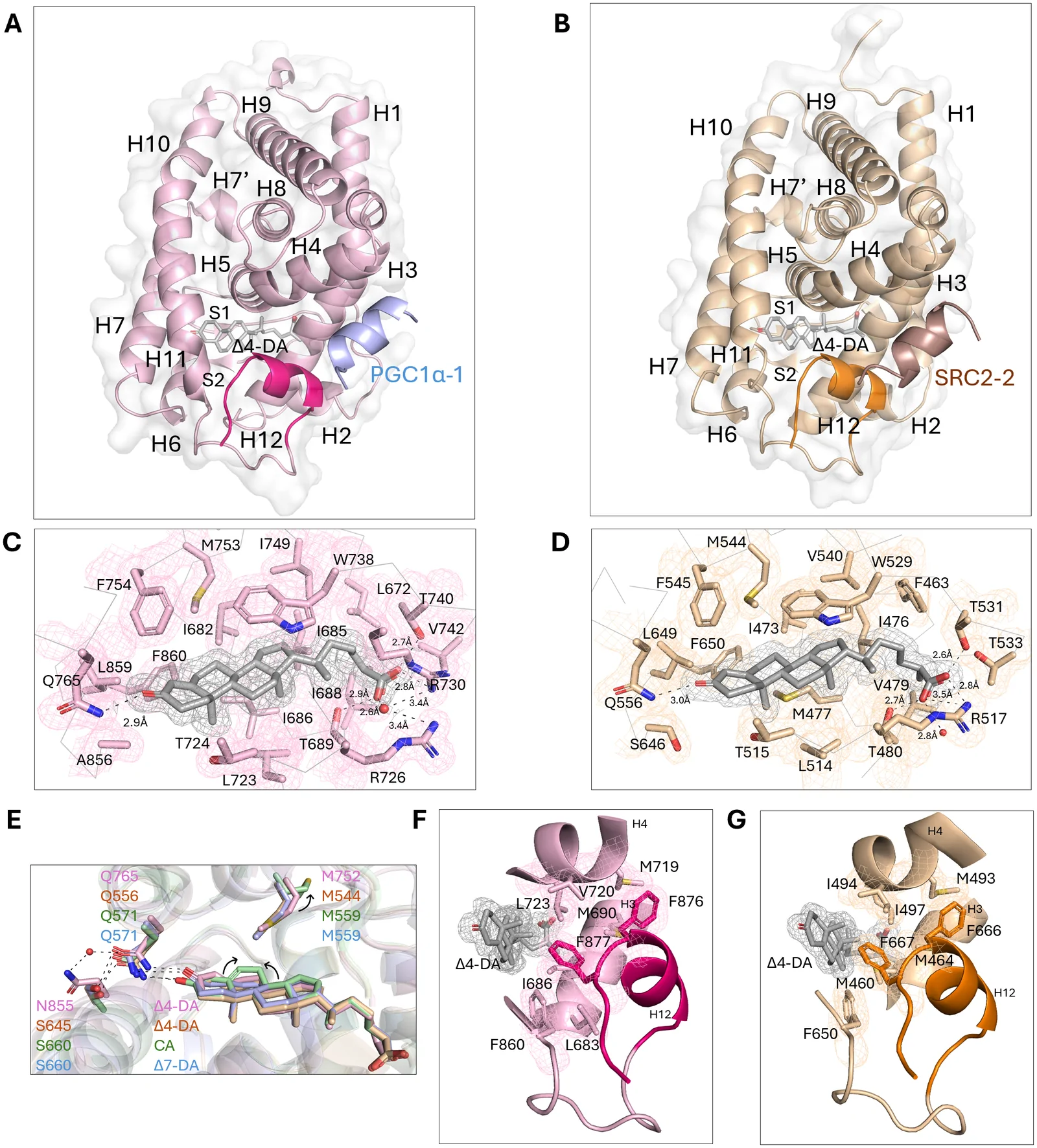
Structural analysis of *Brugia malayi* (*Bma*) *and Haemonchus contortus* (*Hco*) DAF-12 ligand binding domains (LBDs). **A. and B.** Overall structures of *Bma*DAF-12 LBD (light pink) complexed with ∆4-DA (grey) and the coactivator peptide PGC1α-1(blue) (A) and of *Hco*DAF-12 LBD (light orange) complexed with ∆4-DA (grey) and the coactivator peptide SRC2-2 (brown) **(B)**, both in cartoon representation. Helices H12 are highlighted in pink and orange, respectively. **C. and D.** Details of *Bma*DAF-12 (C) and *Hco*DAF-12 (D) ligand binding pockets bound to ∆4-DA. The surrounding amino acids are colored in pink (C) and light orange **(D)**, respectively. The 2*F*_*0*_*-F*_*C*_ electron density maps contoured at 1.4 σ are shown for the ∆4-DA and the surrounding amino acids in the corresponding colors. H-bonding of amino acids and water involved in interactions with the C3-keto group and the C27-carboxyl group of ∆4-DA are shown in black dashes and distances are labeled. **E.** Close-up view of the superimposed crystal structures of *Bma*DAF-12/∆4-DA (pink), *Hco*DAF-12/∆4-DA (orange), *Ace*DAF-12/cholestenoic acid (green; PDB code 3UP3) and *Ace*DAF-12/∆7-DA (cyan; PDB code 3UP0). The comparison illustrates the hydrogen-bonding network involving either the C3-ketone of dafachronic acids or the 3β-hydroxyl group of cholestenoic acid (CA), reveals differences in ligand orientation within the ligand-binding pocket, and highlights the resulting displacement of the indicated methionine residue. **F. and G.** Details of helix H12 active conformation stabilization in the *Bma*DAF-12 (E) and *Hco*DAF-12 **(F)** LBD. Side chains of DAF-12 residues involved in this stabilization are shown as sticks. The 2*F*_*0*_*-F*_*C*_ electron density maps contoured at 1.4 σ are shown for the ∆4-DA as well as the stabilizing residues in the corresponding colors.

To further confirm the mechanism of ligand and coactivator recruitment by *B. malayi* and *H. contortus* DAF-12, we conducted crystallization trials of various DAF-12/ mammalian coactivator-derived peptide/ligand complexes and successfully determined the crystal structures of *Bma*DAF-12 and *Hco*DAF-12 LBDs, in complex with Δ4-DA and PGC1ɑ-1 and SRC2–2, respectively, at resolutions of 1.70 Å and 2.00 Å (Fig 3A and 3B and S2 Table). Both LBDs adopt the canonical nuclear receptor fold, composed of twelve α-helices arranged in a three-layer sandwich and a β-sheet. Of note, instead of the short loop (four to five residues) typically found between helices H7 and H8 in mammalian nuclear receptors, both DAF-12 LBDs feature an additional short α-helix (H7’). This structural element has also been observed in the *Sst*DAF-12 *and Ace*DAF-12 LBDs [14,23], appearing unique and specific to this nuclear receptor. Structural superposition of the *Bma*DAF-12 and *Hco*DAF-12 LBDs yields a root mean square deviation (RMSD) of 0.946 Å over all Cα atoms, indicating a high degree of structural conservation between the two proteins (S5 Fig). Both structures also exhibit strong similarity to previously determined structures of the *Sst*DAF-12 and *Ace*DAF-12 LBDs (S5 Fig), further supporting the conserved architecture of this receptor across parasitic nematodes.

The ligand binding pocket (LBP) volumes of *Bma*DAF-12 and *Hco*DAF-12 were estimated using KVFinder-web [27] (S3 Table). Consistent with previous observations for other DAF-12 orthologs, the LBPs of around 700 Å³ are relatively small in size and fall within the range observed for human nuclear receptors such as VDR, LXR, and FXR, which are amongst the mammalian nuclear receptors that are most closely related to the DAF-12s. Both dafachronic acids have a molecular volume estimated by Chimera X [28] around 420 Å³, showing an occupancy of about 60% of the LBP. The structures of *Bma*DAF-12 and *Hco*DAF-12 LBDs revealed a conserved ligand binding mode, shared with the *Ace*DAF-12 and *Sst*DAF-12 LBDs [14,23] (Fig 3C and 3D). The C3-ketone group of the ligand forms a hydrogen bond with a highly conserved glutamine residue (Q765 in *Bma*DAF-12 and Q556 in *Hco*DAF-12), similar to what was observed with Q571 and Q637 in *Ace*DAF-12 *and Sst*DAF-12, respectively [14,23]. As previously proposed, the length of the alkyl chain in Δ4-DA is critical for positioning the C27 carboxyl group within the LBP. In *Hco*DAF-12, this carboxyl group forms three H-bonds with T480, T531, and R517 (Fig 3D), whereas, in *Bma*DAF-12, it establishes direct hydrogen bonds with the corresponding threonine residues (T689 and T740) and water-mediated interactions with R726 and R730 (Fig 3C). Additionally, Δ4-DA is stabilized by a network of hydrophobic interactions on both sides of its sterol backbone, involving several conserved hydrophobic residues (Fig 3C and 3D and S4 Table), further supporting a conserved mechanism of ligand recognition among DAF-12 orthologs.

Comparison of the structures of *Bma*DAF-12 and *Hco*DAF-12, both in complex with Δ4-DA, with the published structures of *Ace*DAF-12 bound either to cholestenoic acid (PDB code 3UP3) [23] or to Δ7-DA (PDB code 3UP0) [23], suggests two possible explanations for the preferential binding of DAF-12 to dafachronic acids over cholestenoic acid [16]. First, dafachronic acids contain a C3 ketone (3-keto group), whereas cholestenoic acid carries a 3β-hydroxyl group at this position. This substitution alters both hydrogen-bonding properties and local electronic geometry, as carbonyl groups are generally stronger and more directional hydrogen-bond acceptors than hydroxyl groups, while also being less conformationally flexible. In the crystal structures, a glutamine residue (Q765, Q556, Q571 in *Brugia malayi*, *Haemonchus contortus*, and *Ancylostoma ceylanicum*, respectively) positions its terminal NH2 group toward the ligand C3 substituent and acts as a hydrogen-bond donor (Fig 3E). Interaction with the carbonyl group of dafachronic acids is therefore expected to be more favorable than with the hydroxyl group of cholestenoic acid, likely contributing to the higher affinity of dafachronic acids for DAF-12. The orientation of this glutamine is further stabilized by hydrogen-bonds: directly with a serine residue in *Hco*DAF-12 and *Ace*DAF-12 (S645 and S660, respectively), and through a water-mediated hydrogen bond with an asparagine residue (N855) in *Bma*DAF-12 (Fig 3E). Notably, this glutamine is highly conserved among DAF-12 proteins (Figs 5 and S6), except in a small subset that may display distinct ligand preferences. Second, superposition of dafachronic acids and cholestenoic acid in the different DAF-12 ligand binding pockets reveals an approximately 1 Å shift of the first two steroid rings (Fig 3E). In *Bma*, *Hco* and *Ace*DAF-12, a highly conserved methionine residue (M753, M544 and M559, respectively) forms hydrophobic contacts with dafachronic acids. In contrast, in the *Ace*DAF-12-cholestenoic acid structure, the ligand shift displaces this methionine away from the ligand binding pocket (Fig 3E), potentially weakening hydrophobic interactions and reducing ligand affinity. Together, the C3 ketone of dafachronic acids and the positioning of the ligands in the LBP of DAF-12 may account for the higher activity of dafachronic acids for DAF-12 relative to cholestenoic acid.

**Fig 4:**
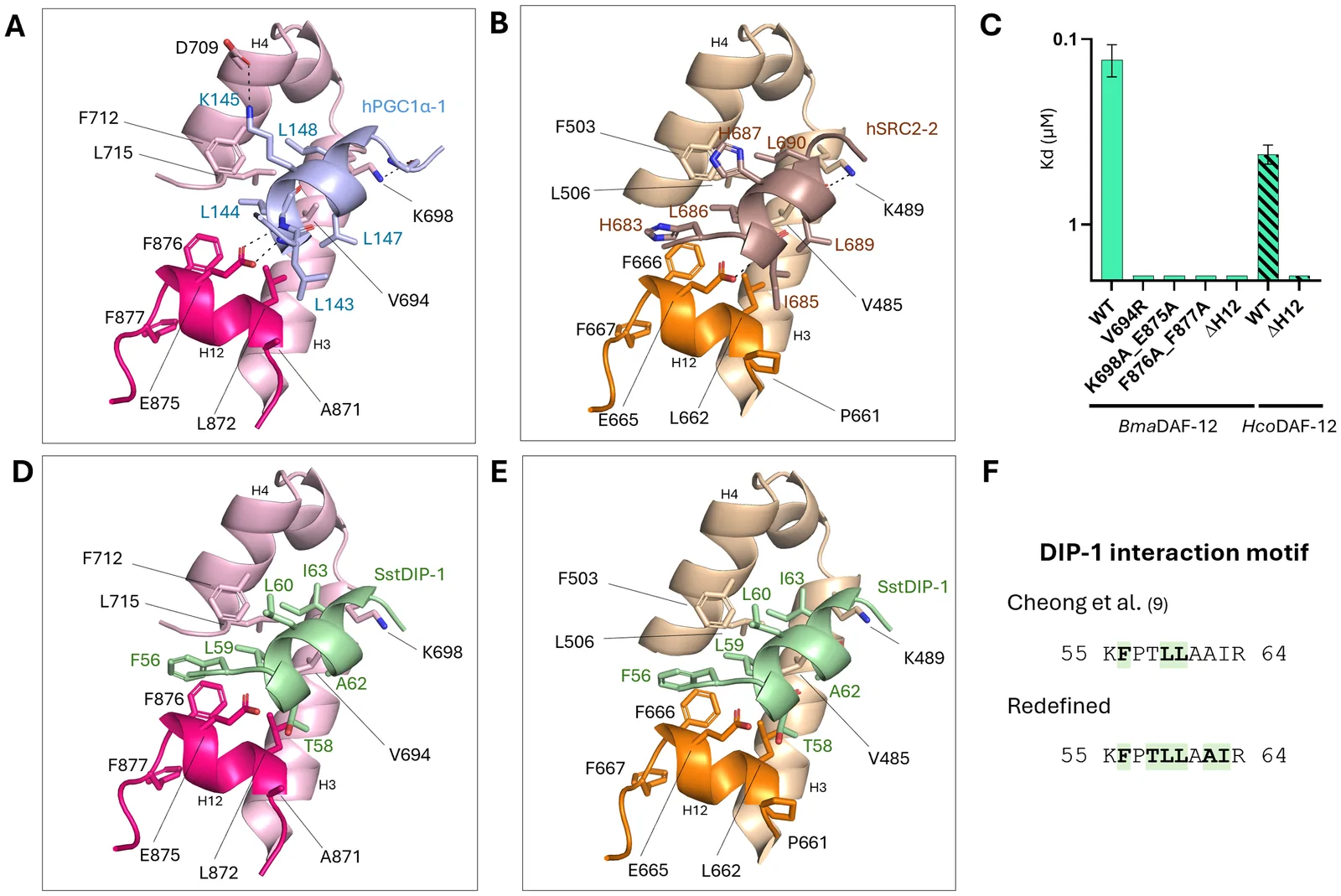
Coactivator binding to the *Brugia malayi* (*Bma*) and *Haemonchus contortus* (*Hco*) DAF-12 ligand binding domains (LBDs). **A. and B.** Close-up view of the *Bma*DAF-12 LBD-PGC1α-1 (A) and *Hco*DAF-12 LBD-SRC2-2 (B) interfaces from the crystal structures of the complexes (presented in this paper). Residues involved in the interaction are shown as sticks and labeled. H-bondings are shown as black dashes. **C.** Affinities of wild-type (WT) and mutant *Bma*DAF-12 (green) and of *Hco*DAF-12 and *Hco*DAF-12∆H12 (hatched green) for the DIP-1 peptide. Dissociation constants (Kd in µM) were derived from fluorescence anisotropy experiments. **D and E.** Close-up view of the *Bma*DAF-12 LBD-DIP-1 (D) and *Hco*DAF-12 LBD-DIP-1 (E) interfaces from AlphaFold models of the complexes generated using the Alphafold3 server [29]. Residues involved in the interaction are shown as sticks and labeled. The DIP-1 interaction motif originally defined by Cheong and al. [13] is shown together with the refined motif identified in the present study based on structural analysis. **F.** Refined definition of the DIP-1 interaction motif. Residues highlighted in green correspond to the minimal interaction motif, whereas residues in bold indicate residues making additional contacts.

**Fig 5:**
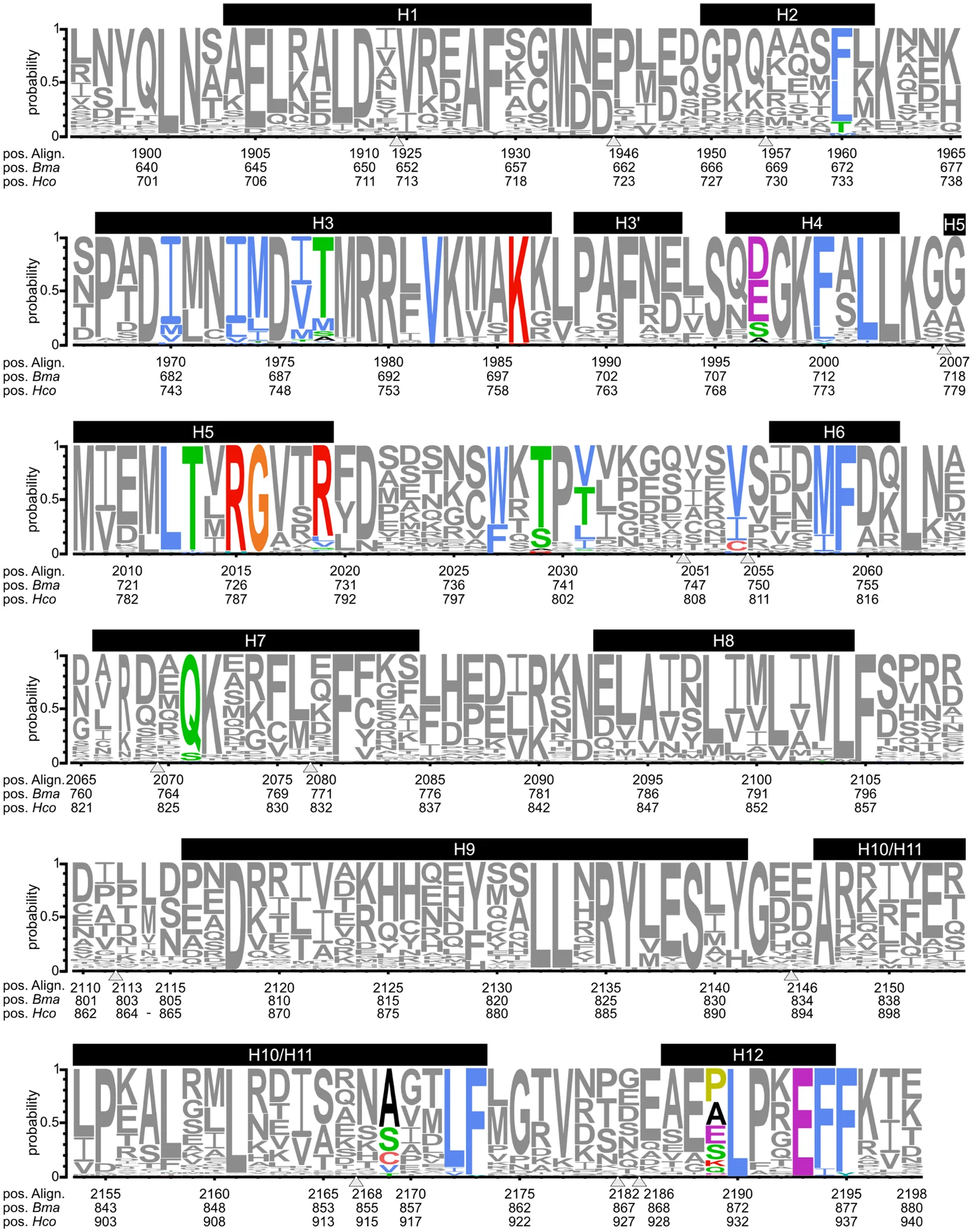
Sequence conservation within DAF-12 ligand-binding domains (LBDs). Sequence logos were generated from positions 1897 to 2198 (corresponding to the LBD) of the untrimmed multiple sequence alignment. Colored residues indicate amino acids experimentally identified as contributing to ligand and coactivator binding, and residues marked by a red asterisk are only involved in coactivator binding. Black horizontal bars refer to secondary structure elements (helices H1 to H12). Arrowheads mark regions that were masked in the alignment (see S6 Fig for the complete alignment).

The ligand Δ4-DA makes direct hydrophobic contacts with F860 in *Bma*DAF-12 and F650 in *Hco*DAF-12, and these phenylalanines in turn create stabilizing π-π stacking interactions with *Bma*DAF-12 F877 and *Hco*DAF-12 F667 in helix H12 (Fig 3F and 3G). These interactions along with additional hydrophobic contacts between Δ4-DA and surrounding residues within the LBP help stabilize helix H12 in its active conformation, a key structural requirement for coactivator peptide recruitment. Coactivator peptides are anchored to the LBDs primarily through hydrophobic contacts involving the leucine residues of their conserved LxxLL motifs (Fig 4A and 4B). These leucines engage a hydrophobic groove on the AF-2 surface, formed by helices H3, H4, and H12. Key interacting residues include a valine on helix H3 (V694 in *Bma*DAF-12 and V485 in *Hco*DAF-12), a phenylalanine (F712 in *Bma*DAF-12 and F503 in *Hco*DAF-12) and a leucine (L715 in *Bma*DAF-12 and L506 in *Hco*DAF-12) on helix H4, and a phenylalanine on helix H12 (F876 in *Bma*DAF-12 and F666 in *Hco*DAF-12). In addition, electrostatic interactions occur between the peptide and a characteristic charged clamp formed by a lysine on helix H3 (K698 in *Bma*DAF-12 and K489 in *Hco*DAF-12) and a glutamic acid on helix H12 (E875 in *Bma*DAF-12 and E665 in *Hco*DAF-12) that stabilizes the coactivator complex. These key residues are also conserved in the DAF-12 orthologs [14,23], indicating a broadly conserved coactivator binding mechanism among DAF-12, similar to that of mammalian nuclear receptors.

### Additional motif-specific interactions between nematode DAF-12 and coactivator peptides

Crystal structures of *Bma*DAF-12 and *Hco*DAF-12 in complex with coactivator-derived peptides reveal motif-specific interactions that extend beyond the canonical LXXLL binding mode. Notably, an electrostatic interaction occurs between lysine K145 in the LKKLL motif of PGC1α-1 and aspartic acid D709 of *Bma*DAF-12 (Fig 4A). A comparable interaction may also contribute to binding of Δ4-DA-bound DAF-12 to SRC1–3 and SRC2–3 peptides (Table 1), which harbor a similar LK/RXLL motif. In the *Hco*DAF-12 complex structure, a π–π stacking is detected between histidine H687 from the LHRLL motif of SRC2–2 and phenylalanine F503 of *Hco*DAF-12, with a distance of about 3.3 Å between the parallel aromatic rings (Fig 4B). This interaction may similarly participate in the recognition of the SRC2–2, SRC1–2, and SRC3–2 peptides by Δ4-DA-bound DAF-12 (Table 1). All motifs interacting with *Bma*DAF-12 and *Hco*DAF-12 also feature a hydrophobic residue immediately preceding the LXXLL motif, yielding a [I/L/M/T]LXXLL consensus (Table 1). This residue (L143 in PGC1α-1 and I685 in SRC2–2) faces hydrophobic residues from helix H12 (alanine A871 and leucine L872 from *Bma*DAF-12 and proline P661 and leucine L662 from *Hco*DAF-12), creating additional hydrophobic contacts and further stabilizing the coactivator peptide on the DAF-12 activation surface (Fig 4A and 4B). Furthermore, the crystal structure of *Hco*DAF-12 bound to SRC2–2 reveals T-shaped CH–π interactions between the histidine H683, at position -3 relative to the first leucine of the LXXLL motif, and two aromatic residues, F503 and F666, of *Hco*DAF-12 (Fig 4B). Of note, these two phenylalanine residues are conserved in *Bma*DAF-12 (F712 and F876), but not present in mammalian nuclear receptors (Fig 2A). Consistent with these structural observations, the SRC1–1 peptide, which lacks these additional interacting residues, showed no detectable interaction with either DAF-12 receptor.

To evaluate the contribution of individual coactivator peptide residues to DAF-12 binding, we introduced targeted point mutations into the SCR1–1 peptide and quantified binding affinities using fluorescence anisotropy assays (Table 1). Substitution of the lysine at position -1 of the LXXLL motif with a hydrophobic residue (K632L) as well as the double substitution of residues at positions -3 and +1 of the LXXLL motif into histidine (S630H/V634H), resulted in weak but detectable interactions of the corresponding peptides with *Bma*DAF-12 and *Hco*DAF-12 LBDs. In contrast, substitution of the valine within the LVQLL motif by a lysine (V634K) did not induce an interaction of the peptide with the DAF-12 LBDs. Strikingly, combining the three substitutions to generate the S630H/K632L/V634K peptide, which conforms to a HXLLKXLL motif similar to that of the SRC1–3 peptide, yielded high-affinity binding with dissociation constants in the presence of Δ4-DA of approximately 100 nM for *Bma*DAF-12 and 771 nM for *Hco*DAF-12. These mutagenesis experiments thus confirm the critical contribution of residues flanking or within the LXXLL core motif to coactivator binding to DAF-12. On the contrary, these substitutions on SRC1–1 did not induce any interaction with the mammalian nuclear receptors LXRβ and RXRα (S4 Fig), suggesting a specificity towards DAF-12. Collectively, the structural and biochemical analyses uncover novel, motif-specific contacts beyond the canonical LXXLL interaction, providing mechanistic insights into the sequence determinants of DAF-12 coactivator binding in parasitic nematodes.

### A conserved interaction mode between *B. malayi* and *H. contortus* DAF-12 LBDs and *S. stercoralis* DIP-1 and a new definition of its interaction motif

To assess whether the recruitment of *S. stercoralis* DIP-1, the sole coactivator identified in parasitic nematodes, follows a canonical mechanism, we monitored the binding of the DIP-1 peptide spanning residues V49 to K65 to wild-type *Bma*DAF-12 and its mutants (Figs 4C and S3D). Although *Sst*DIP-1 contains a variant motif (FXXLL rather than LXXLL), the same DAF-12 mutations that disrupted binding to the mammalian coactivator peptides (Fig 2D) also abolished interaction with *Sst*DIP-1, supporting the notion that DAF-12 engages both mammalian and nematode coactivators through a conserved structural mechanism. Similarly, the DAF-12 deletion constructs lacking helix H12 (*Bma*DAF-12ΔH12 and *Hco*DAF-12ΔH12) were not able to interact with the DIP-1 peptide, confirming the role of helix H12 in the interaction. To further investigate the binding of *Sst*DIP-1 to DAF-12, we generated AlphaFold [29] models of the *Bma*DAF-12 and *Hco*DAF-12 LBDs in complex with this peptide and compared them to the experimentally determined structures presented in this study (Fig 4D and 4E). Interestingly, this model suggested that the interaction motif of *Sst*DIP-1 is more consistent with a L_59_XXA I_63_ motif rather than with the initially proposed F_56_XXL L_60_ motif [13]. Leucine L59 and isoleucine I63 play the role of the first and last leucines, respectively, in the classical LXXLL motif by establishing contacts with hydrophobic residues from the AF-2 surface as described above. These observations align with the mutagenesis results reported by Cheong and colleagues [13], which identified the leucine L59 as a critical determinant for *Sst*DIP-1 binding since its mutation into alanine strongly disrupted binding while the L60A mutation has a very limited impact on binding. Similar to what has been observed for other coactivator peptides and in mutagenesis studies of hSRC1–1, the residue immediately preceding the LXXAI (or LXXLL) motif can contribute to binding through additional hydrophobic interactions with DAF-12, thereby stabilizing the peptide on the DAF-12 activation surface. In *Sst*DIP-1, this residue corresponds to T58, which forms hydrophobic contacts with L872 in *Bma*DAF-12 and L662 in *Hco*DAF-12. Consistent with this interpretation, Cheong and colleagues showed that the T58A mutation decreases *Sst*DIP-1 affinity for DAF-12 [13]. A similar observation was made with the K632L hSRC1–1 mutant, where substitution with a hydrophobic residue allowed the interaction (Table 1). Likewise, an aromatic residue at position -3 relative to the LXXAI (or LXXLL) motif also contributes to binding through interactions with aromatic residues on DAF-12, although its contribution appears less critical. In *Sst*DIP-1, this residue corresponds to F56, which appears to contribute to the interaction in a manner similar to that of histidine H683 in the SRC2–2 peptide, forming a T-shaped CH–π interaction with F712 (H4) and F876 (H12) from *Bma*DAF-12 and with F503 (H4) and F666 (H12) from *Hco*DAF-12 (Fig 4D and 4E). Consistent with this model, Cheong and colleagues showed that the mutation of F56 to alanine only partially weakens the interaction with DAF-12 [13]. Similar effects were observed when comparing the S630H/V634H and S630H/K632L/V634K hSRC1–1 mutants (Table 1). To conclude, this detailed structural analysis allowed us to re-define the interaction motif of *Sst*DIP-1 as a FXXLXXAI motif (Fig 4F) suggesting that the interaction motifs of nematode coactivators are similar, but not identical, and thus more diverse than their mammalian counterparts.

### DAF-12 LBD is conserved in nematodes from clades III, IV, and V

To determine whether *B. malayi* and *H. contortus* DAF-12 residues implicated in coactivator recruitment are conserved across nematodes, we performed a comprehensive bioinformatic analysis of DAF-12 sequences. We surveyed all nematode genomes available in WormBase [30] and identified DAF-12 orthologs exclusively in species belonging to clades III, IV, and V. A phylogenetic tree based on an alignment of 118 full-length DAF-12 sequences (S6 Fig) closely recapitulated nematode taxonomy, with sequences clustering according to their respective clades. We next examined sequence conservation within the DAF-12 LBDs (Fig 5). A sequence logo analysis revealed a high degree of conservation, particularly in regions corresponding to helices H3, H4, H5, and H12. In contrast, sequence conservation was significantly lower in loop regions. Overall, DAF-12 sequences were most highly conserved among clade III nematodes, whereas greater, though still limited, sequence variability was observed within clade IV and clade V nematodes (S7 Fig).

With respect to residues involved in ligand binding, all the hydrophobic residues lining the LBP were markedly conserved in terms of their hydrophobic character. The arginine corresponding to R726 in *B. malayi* (and R517 in *H. contortus*), which is critical for coordinating the carboxyl group of dafachronic acids, was strictly conserved in all DAF-12 sequences (Fig 5). By comparison, residues that play a more auxiliary role in ligand recognition, such as arginine R730 and threonine T740 (*B. malayi* numbering*)*, displayed some minor variability (S7 Fig). Similarly, the glutamine residue corresponding to Q765 in *B. malayi* (Q556 in *H. contortus*), which contributes to binding of the C3-ketone group of the ligand, was strongly conserved across clades. Together, these observations suggest that dafachronic acids are likely to serve as common ligands for DAF-12 across different nematode clades.

Regarding residues involved in coactivator binding, all positions described above to be critical for the recruitment of coactivator-derived peptides were strictly conserved across the DAF-12s from the three clades, with two notable exceptions. First, the aspartic acid corresponding to D709 in *B. malayi*, which forms an electrostatic interaction with K145 of PGC1α-1 (Fig 4A) was highly conserved in clade III nematodes, but showed a notable variability in clades IV and V (S7 Fig). Similarly, alanine A871 in *B. malayi* DAF-12, which faces a hydrophobic residue in the coactivator-derived peptide, was highly conserved in clade III nematodes, but was more variable in clades IV and V, including substitutions by a charged residue (S7 Fig). This suggests that DAF-12 proteins might exhibit clade-specific differences in coactivator preference. Together, these observations on DAF-12 sequences from different nematode clades support a conserved interaction mechanism with coactivators and suggest that residues flanking or within the LXXLL motifs may contribute to the specificity of coactivator binding to DAF-12 and that this specificity might vary between different nematode clades.

## Discussion

In this study, we focused on the structure of DAF-12 receptors from two parasitic nematodes of major biomedical and veterinary importance: *B. malayi*, a filarial nematode causing several NTDs with a huge impact on human health, and *H. contortus*, a gastrointestinal nematode of wild and domesticated ruminants that is highly pathogenic and economically significant.

The in-depth structural analysis of DAF-12 from *B. malayi* and *H. contortus* in complexes with transcriptional coactivator-derived peptides combined with mutagenesis experiments and a comprehensive bioinformatic analysis of DAF-12 sequences allowed us to identify key structural features shared across all DAF-12 orthologs. These include the residues lining the ligand binding pocket as well as the canonical charge clamp formed by K698 and E875 in *B. malayi* (K489 and E665 in *H. contortus*), and the hydrophobic groove formed by residues from H3, H4, and H12, both involved in coregulator binding. Comparison with the closest mammalian nuclear receptors, namely VDR, FXR, and LXR, revealed additional DAF-12-specific features that are not conserved in mammalian nuclear receptors, within the coactivator binding surface. Notably, the strong conservation of F712 and F876 in *B. malayi* (F503 and F666 in *H. contortus*) suggests nematode-specific interaction determinants that may facilitate selective recruitment of nematode transcriptional coactivators. Such differences could potentially be exploited to develop compounds that selectively modulate nematode DAF-12 activity while minimizing cross-reactivity with mammalian nuclear receptors. In addition, we confirmed the agonist activities of Δ4- and Δ7-DAs, two known DAF-12 ligands [7,12,31], in promoting the recruitment of coactivators, in particular that of *S. stercoralis* DIP-1 [13], to *B. malayi* and *H. contortus* DAF-12. Altogether, these results support a conserved mechanism of DAF-12 regulation in parasitic nematodes, in line with the essential role of this nuclear receptor in nematode development [9–15].

With the exception of *Sst*DIP-1 that is found only in the parasitic nematode family Strongyloididae [13], no transcriptional coactivators have been described in nematodes, which limits our ability to fully characterize the regulatory mechanisms governing DAF-12 and other nuclear receptors. Nevertheless, our current findings provide a framework for the identification and characterization of DAF-12 coactivators in these species and for investigating their specific roles in DAF-12-mediated transcriptional regulation. Specifically, our data allowed us to define a conserved interaction motif in coactivator-derived peptides that interact with DAF-12 and are characterized by the consensus sequence [F/H]X[L/I/T/M]LXX[A/I/L][I/L]. Given that complete genome sequences of a number of different nematodes are available in WormBase [30], this motif provides a basis for a systematic *in silico* identification of potential DAF-12 coactivators. To further refine candidate selection, priority should be given to proteins containing two to three such motifs, in accordance with the architecture of mammalian nuclear receptor coactivators [32]. Supporting the utility of this approach, a search within the complete *Strongyloides stercoralis* proteome using FuzzPro (European Bioinformatics Institute, Cambridgen UK) identified 565 proteins containing at least one motif match. Of these, 532 proteins contained a single occurrence, 32 contained two occurrences, and one contained three occurrences. Notably, *Sst*DIP-1 was recovered among the identified candidates. Although the motif alone is not sufficiently selective to define *bona fide* coactivators, the successful retrieval of *Sst*DIP-1 provides a proof of principle that this strategy can facilitate the discovery of novel coregulators in nematode proteomes of interest. Importantly, candidates conserved between parasitic nematodes and *C. elegans* could be readily prioritized for functional validation in *C. elegans* using established genetic approaches, such as RNAi-mediated knockdown, to assess their roles in DAF-12-dependent processes including dauer entry and exit.

DAF-12 is evolutionarily conserved across nematodes, particularly within its DNA-binding and ligand-binding domains. Antebi and colleagues identified several mutations in *C. elegans* DAF-12 that result in dauer formation defects [33]. In particular, the R564C and R564H substitutions produce a dauer-constitutive (Daf-c) phenotype, suggesting disruption of normal ligand-dependent regulation of DAF-12. Notably, this arginine residue is strictly conserved among nematode DAF-12 orthologs (Figs 5 and S6), supporting its critical functional role. Analysis of the available DAF-12 crystal structures reveals that R564 (corresponding to R692, R483, and R521 in *Brugia malayi*, *Haemonchus contortus*, and *Ancylostoma ceylanicum*, respectively) is located within helix H3. Although this residue does not establish direct contacts with the ligand (whether dafachronic acids or cholestenoic acid), structural inspection suggests that it may contribute to stabilization of the H1-H2 loop through hydrogen-bonding interactions. This loop is positioned in close proximity to the ligand carboxylate group and contributes to the architecture of the LBP. Consequently, substitution of R564 by cysteine or histidine may disrupt this interaction network and indirectly impair ligand binding. Antebi et al. also described the neighboring M562I missense mutation, corresponding to the class 3 allele *m25*, which confers a partially Daf-d phenotype characterized by mosaic dauer formation [33]. Inspection of the crystal structures indicates that this methionine lies near both the LBP and the activation helix H12. Given the central role of H12 in coactivator recruitment, replacement of methionine by the more β-branched isoleucine could alter local packing interactions and could affect the positioning or dynamics of H12. Such structural perturbations may ultimately influence receptor activity. Further structural and biophysical characterization of these DAF-12 variants will be required to definitively establish the molecular consequences of these substitutions. Nevertheless, the detailed analysis of DAF-12 crystal structures from parasitic nematodes provides a valuable framework for predicting and rationalizing the functional effects of analogous mutations in *C. elegans*.

Previous studies have established that DAF-12 is required in *C. elegans* both to enter into and exit from the dauer diapause, a developmental stage that corresponds to the infective L3 stage in many parasitic nematodes [7,8]. Our findings demonstrate that the mechanism of activation of DAF-12 is highly conserved across parasitic nematodes, underscoring the role of this nuclear receptor in parasite development. This conservation also suggests that targeting DAF-12 signaling represents a promising therapeutic strategy, distinct from current anthelmintic drug targets, for the control of parasitic nematode infections affecting humans, animals, and plants. The structural insights provided by our two newly determined DAF-12 crystal structures provide a valuable framework for the rational design of compounds that selectively target DAF-12 activity, with potential therapeutic applications against parasitic nematodes. In principle, these structures could be exploited for structure-based virtual screening to identify compounds capable of occupying the LBP and competing with endogenous dafachronic acids, thereby preventing coactivator recruitment and subsequent DAF-12 activation. However, several challenges are likely to complicate this strategy. First, the chemical synthesis and derivatization of dafachronic acids are particularly challenging [34], due to the structural complexity of these ligands. Second, our structural data indicate that the LBP is highly optimized for dafachronic acid recognition in agreement with their strong binding affinity, limiting opportunities for the development of competitive antagonists. Consequently, successful identification of such compounds will likely require screening of very large and chemically diverse libraries. An alternative and potentially more tractable strategy would be to target the protein–protein interactions required for DAF-12 transcriptional activity. The crystal structures reported here, solved in complex with coactivator peptides, provide detailed information on the molecular determinants governing coactivator recognition and could guide the design of molecules that disrupt these interactions. Such compounds could include small molecules, peptides, peptidomimetics or antibodies [35] capable of modulating co-regulator recruitment. Conventional high-throughput screening approaches could be applied to identify such modulators, while emerging hit-discovery technologies, including DNA-encoded libraries (DELs) [36,37], offer particularly attractive opportunities to explore large chemical spaces. In conclusion, as such, this study could significantly contribute to future anthelmintic drug discovery efforts.

## Materials and methods

### Ligands and peptides

Δ4-dafachronic acid (Δ4-DA) and (25*S*) Δ7-dafachronic acid (Δ7-DA) were purchased from Cayman and ChemCruz, respectively. The fluorescent peptides labelled with FITC at their N-terminal and listed in Table 1 were purchased from Proteogenix. The unlabelled peptides PGC1α-1 (EEPSLLKKLLLAPA) and SRC2–2 (KHKILHRLLQDSS) used for crystallization were purchased from Ezbiolab.

### Protein purification

DNA sequences corresponding to *Bma*DAF-12 LBD (from L636 to T880, Uniprot entry A0A4E9F2L4), *Hco*DAF-12 LBD (from L428 to E670, Uniprot entry A0A7I4Z2W0) and *Hco*DAF-12 LBD (C538S, C592S, C610S, C646S), *Bma*DAF-12ΔH12 (from L636 to G862), and *Hco*DAF-12ΔH12 (from L428 to G652) have been optimized for *Escherichia coli* expression using the Integrated DNA Technologies (IDT) tool and were purchased at IDT. The sequences were cloned into the pDB-His-TXR-3C vector and expressed using the *E. coli* BL21(DE3) cellular system.

Cells were grown at 37°C in LB medium supplemented with 50 µg/mL kanamycin until OD600 reached about 0.6. Expression of T7 polymerase was induced by addition of isopropyl-β-d-thiogalactoside (IPTG) to a final concentration of 0.5 mM for *Bma*DAF-12 LBD and of 1mM for *Hco*DAF-12 LBD. After an additional incubation of 24 hours at 16°C, cell cultures were harvested by centrifugation at 6,000 g for 20 minutes. The cell pellet was resuspended in buffer A (20 mM Tris-HCl pH 7.5, 150 mM NaCl for *Bma*DAF-12 and *Bma*DAF-12ΔH12; 20 mM Tris-HCl pH 7.5, 200 mM NaCl, 0.2% IGEPAL for *Hco*DAF-12 and 0.5% IGEPAL for *Hco*DAF-12ΔH12), supplemented with a protease inhibitor mixture (complete, mini, EDTA-free tablet) and 10 µg/mL lysozyme. The suspension was then lysed by sonication and centrifuged at 40,000 g and 6°C for 30 minutes. The supernatant was loaded onto a 5 mL Ni^2+^-affinity column, preequilibrated with buffer A containing 10 mM imidazole, using the Akta purifier system. The column was washed with 20 column volumes (CV) of buffer A containing 10 mM imidazole and 20 CV of buffer A containing 20 mM imidazole. Bound proteins were incubated overnight at 4°C with an excess of PreScission protease to cleave the His-TRX tag. Unbound protein was eluted with buffer A containing 30 mM imidazole. The protein was further purified using a Superdex 75 16/60 gel filtration column preequilibrated with buffer C (20 mM Tris-HCl pH 7.5, 150 mM NaCl, 5% (v/v) glycerol, 2 mM DTT for *Bma*DAF-12 and *Bma*DAF-12ΔH12; 20 mM Tris-HCl pH 7.5, 150 mM NaCl, 5% (v/v) glycerol, 1 mM DTT for *Hco*DAF-12 and 50 mM Tris pH8, 500 mM NaCl, 5% (v/v) glycerol for *Hco*DAF-12ΔH12). The protein-containing fractions were pooled and concentrated using Amicon-Ultra 10000 MXCO centrifugal filter units (Millipore).

### Nano-differential scanning fluorimetry (Nano-DSF)

Nano-differential scanning fluorimetry (Nano-DSF) experiments were performed on a Tycho NT.6 instrument (NanoTemper) to analyze the thermal stability of the proteins. Protein samples were prepared at 5 µM for *Bma*DAF-12 in SEC buffer (20 mM Tris-HCl pH 7.5, 150 mM NaCl, 5% (v/v) glycerol, and 2 mM DTT) and at 20 µM for *Hco*DAF-12 in SEC buffer (20 mM Tris-HCl pH 7.5, 150 mM NaCl, 5% (v/v) glycerol, and 1 mM DTT). Samples were prepared in the presence of one and three molar equivalents of ligands initially prepared at 20 mM in DMSO. For the reference samples, DMSO alone was added to a final concentration identical to the one in the presence of the ligand. Samples were loaded in a 10 µL capillary and submitted to a temperature gradient starting at 35°C and finishing at 95°C. The ratio of emission intensities at 350 nm and 330 nm was measured, the resulting melting curves were generated by plotting the first derivative of this ratio against temperature, and the temperature of inflection (Ti) was defined as the maximum of this derivative curve.

### Steady-state fluorescence anisotropy

Fluorescent anisotropy assays were performed using a Safire microplate reader (TECAN) with the excitation wavelength set at 470 nm and emission measured at 530 nm for FITC-labeled peptides. The buffer solution for the assays was 20 mM Tris-HCl pH 7.5, 150 mM NaCl, 5% (v/v) glycerol, and 2 mM DTT. The measurements were initiated at a high protein concentration (10 µM of *Bma*DAF-12 or *Hco*DAF-12), and the protein sample was then repeatedly diluted 2-fold with buffer solution. For each point of the titration curve, the protein sample was mixed with fluorescent peptide (5 nM final concentration) and 20 µM of ligand (two molar excess relative to the highest concentration of protein) for ligand-bound measurements. The reported data are the average of at least three independent experiments, and error bars correspond to standard deviations. Kd values were deduced by non-linear sigmoidal analysis using GraphPad (Prism).

### Cell-based transactivation assays

NIH3T3 cells (ATCC) were cultured in DMEM (Dulbecco’s modified Eagle’s medium) with L-glutamine supplemented with 10% (v/v) FBS containing 100 U/mL penicillin and 100 μg/mL streptomycin. To perform the cell-based transactivation assays, 12.5 × 10^3^ NIH3T3 cells were seeded in white 96-well plates with transparent bottom. After 24 hours, the cells were transiently transfected in 125μL serum-free DMEM with 50 ng of pFN26A_DAF-12-LBD constructs bearing a Renilla luciferase gene used for normalization and 50 ng of pGL4.35 plasmid bearing the luciferase reporter gene under the control of the Gal4 response element (UAS, upstream activation sequence) (Promega) using 0.3 μL of TransIT-X2 transfection reagent (Mirus Bio) in 10μL of Opti-MEM. Serum-free medium was replaced 5 hours later by 200 μL of complete medium with ligands or vehicle control. The final concentration of DMSO was maintained at 0.1% in each well. After an incubation of 24 hours, the cells were lysed and luciferase and renilla activities were successively measured using the Dual-Glo luciferase assay system (Promega) with a FLUOstar OMEGA microplate reader (BMG Labtech). In order to draw a dose response curve, we first calculated the ratio of luminescence from the luciferase reporter to luminescence from the renilla reporter for each condition and replicate. The ratio of DAF-12-transfected cells was normalized to the ratio of empty vector-transfected cells that were treated under the same conditions. The curve was fitted using GraphPad Prism 8 under a non-linear regression fit based on a sigmoidal dose-response with variable slope.

### Crystallization and structure resolution

Crystals of *Bma*DAF-12 and *Hco*DAF-12 complexes with ∆4-DA and peptides derived from coactivators (PGC1ɑ-1 and SRC2–2, respectively) were obtained by co-crystallization. 100 nL of protein concentrated at 13.5 mg/mL and 9.6 mg/mL with two molar equivalents of ∆4-DA and two molar equivalents of PGC1ɑ-1 for *Bma*DAF-12 and with three molar equivalents of ∆4-DA and five molar equivalents of SRC2–2 for *Hco*DAF-12 were mixed with 100 nL of precipitant solution (0.2 M ammonium citrate dibasic, 20% (w/v) PEG 3350 for *Bma*DAF-12 and 2.4 M sodium malonate pH 7 for *Hco*DAF-12) and equilibrated against a reservoir of 50 µL of precipitant solution, using the sitting-drop vapor diffusion technique. Crystals appeared in 24 hours. Crystals were mounted from mother liquor onto a cryoloop, soaked in the precipitant solution containing 20% (v/v) glycerol and flash-frozen in liquid nitrogen. Diffraction data were collected at the ID-30B beamline at the European Synchrotron Radiation Facilities (λ = 0.969 Å, 100 K) at 1.70 Å resolution for *Bma*DAF-12 and 2.0 Å resolution for *Hco*DAF-12. Diffraction data were automatically processed using the Information System for Protein Crystallography Beamlines (ISPyB). The initial phases were obtained by automatic molecular replacement using Phenix.phaser [38] with the Alphafold [29] model of the corresponding complexes. Models were built with Coot [39] and refined with Phenix.refine [38]. Figures were prepared with PyMOL (The PyMOL Molecular Graphics System, Version 3.0 Schrödinger, LLC.).

### Phylogenetic analysis and sequence logos

Full-length amino acid sequences of DAF-12 were recovered from WormBase ParaSite GeneTree WBGT00950000409411. After initial curation, partial sequences (lacking clearly-defined DBD and/or LBD domains) were removed from the analysis. Accession numbers of the retained sequences are provided in S5 Table. An initial amino acid alignment was performed using Clustal Omega [40] (S1 File), which was followed by automated refinement using BMGE [41] (S2 File). The phylogenetic tree was calculated based on the final alignment of 118 sequences using 331 amino acid positions and allowing gaps. Phylogenetic relationships were assessed using the Maximum Likelihood (ML) method as implemented in RAxML [41]. The ML tree was calculated by applying a LG matrix [42], allowing for a proportion of invariable sites (+I) and a discrete Gamma distribution (+G4). The robustness of each node of the resulting tree was assessed by rapid bootstrap analyses (with 1,000 pseudoreplicates). The tree was visualized using iTOL [42] rooted with the *Plectus sambesii* DAF-12 sequence. The image output of the alignment was generated using Unipro UGENE [42], with a Clustal X coloring palette (blue for hydrophobic residues, red and magenta for positively and negatively charged residues respectively, green for polar residues, pink for cysteines, orange for glycines, yellow for prolines, cyan for aromatic residues, and grey for unconserved residues) and a sequence order based on the results of the phylogenetic tree. Sequence logos for the sequence stretches corresponding to the LBD of *B. malayi* and *H. contortus* were calculated for the entire untrimmed alignment using WebLogo3 [42].

## Supporting information

S1 Fig Biophysical characterization of the *Brugia malayi* (*Bma*) and *Haemonchus contortus* (*Hco*) DAF-12 ligand-binding domains (LBDs). **A. and B.** Size exclusion chromatography (SEC) chromatograms of *Bma*DAF-12 LBD (A) and *Hco*DAF-12 LBD (B) with respective 10% SDS-PAGE Bis-Tris gel of the protein after the final purification step. **C.** Alignment of *Hco*DAF-12 and *Ancylostoma ceylanicum* (*Ace*) DAF-12 LBD sequences. Secondary structure elements (H, α-helix; B, β-strand) from the crystal structures are indicated. Cysteines that were mutated into serines in both proteins are highlighted in purple. **D.** Overlay of far-UV circular dichroism (CD) spectra of the *Bma*DAF-12 and *Hco*DAF-12 LBDs. Secondary structure elements coming from the deconvolution of these data are indicated (a) and compared to the theoretical values (b). **E.** Mass-weighted dynamic light scattering (DLS) size-distribution profiles for the purified *Bma*DAF-12 and *Hco*DAF-12 LBDs. The deduced hydrodynamic radii are indicated and are in agreement with the size of the monomeric form of the proteins.
(TIF)

S2 Fig Ligand binding to the *Brugia malayi* (*Bma*) and *Haemonchus contortus* (*Hco*) DAF-12 ligand-binding domains (LBDs). **A. and B.** Nano-DSF analysis of ligand binding to the *Bma*DAF-12 (A) and wild-type and mutant *Hco*DAF-12 (B) LBDs. ∆4- and ∆7-dafachronic acids (∆4-DA and ∆7-DA) or DMSO (apo condition) were added in three molar excess to the protein. The first derivative of the ratio between intrinsic fluorescence at 350 nm and 300 nm is plotted against the temperature. The experiments were repeated three times independently. **C. and D.** Native mass spectra of apo *Bma*DAF-12 LBD (C) sprayed at 20 µM, showing one species corresponding to the protein at 28 755 Da (grey circles), and *Bma*DAF-12 in the presence of 50 µM Δ4-DA (D), showing an additional species at 29 170 Da corresponding to one Δ4-DA bound to *Bma*DAF-12 (grey circle with red star).
(TIF)

S3 Fig Titration curves of affinity measurements on the *Brugia malayi* (*Bma*) and *Haemonchus contortus* (*Hco*) DAF-12 ligand-binding domains (LBDs) by fluorescence anisotropy. **A.** Affinity curves for the *Bma*DAF-12 (left) and *Hco*DAF-12 (right) LBDs and human coactivator-derived peptides (Table 1) in the absence of ligand. **B.** Affinity curves for the *Bma*DAF-12 (left) and *Hco*DAF-12 (right) LBDs and human coactivator-derived peptides (Table 1) in the presence of two molar excess of ∆4-DA. **C.** Affinity curves for the *Bma*DAF-12 (left) and *Hco*DAF-12 (right) LBDs and DIP-1-derived peptide in the absence of ligand and in the presence of two molar excess of ∆4-DA and ∆7-DA. **D.** Affinity curves for WT and mutant *Bma*DAF-12 LBDs and PGC1α-1 (left) and DIP-1 (right) peptides in the presence of two molar excess of ∆4-DA. **E.** Affinity curves for *Hco*DAF-12 and *Hco*DAF-12ΔH12 LBDs and SRC2–2 (left) and DIP-1 (right) peptides in the presence of two molar excess of ∆4-DA. **F.** Affinity curves for the *Bma*DAF-12 (left) and *Hco*DAF-12 (right) LBDs and WT and mutant SRC1–1 peptides in the presence of two molar excess of ∆4-DA.
(TIF)

S4 Fig Titration curves of affinity measurements on the mammalian LXRβ and RXRα LBDs and WT and mutant SRC1–1 peptides in the presence of two molar excess of agonist ligands (epoxycholesterol and CD3254, respectively), by fluorescence anisotropy. (TIF)

S5 Fig Structural conservation of the DAF-12 ligand-binding domains (LBDs) from different nematodes. **A.** Superimposition of the *Brugia malayi* (*Bma*) DAF-12-∆4-DA-PGC1α-1 (PDB 9TLX, pink) structure with the *Haemonchus contortus* (*Hco*) DAF-12-∆4-DA-SRC2–2 (PDB 9TL4, wheat), the *Strongyloides stercoralis* (*Sst*) DAF-12-∆4-DA-SRC1–4 (PDB 3GYT, orange), and the *Ancylostoma ceylanicum* (*Ace*) DAF-12-∆7-DA-SRC2–3 (PDB 3UP0, green) structures. **B.** Table of calculated root mean square deviation (RMSD) over the Cα between the *Bma*DAF-12-∆4-DA-PGC1α-1, the *Hco*DAF-12-∆4-DA-SRC2–2, the *Sst*DAF-12-∆4-DA-SRC1–4, the *Sst*DAF-12-∆7-DA-SRC1–4, the *Ace*DAF-12-CA-SRC2–3, and the *Ace*DAF-12-∆7-DA-SRC2–3 structures.
(TIF)

S6 Fig Phylogenetic tree of DAF-12 proteins and sequence alignment of the ligand binding domain (LBD) (positions 1880–2207 of the untrimmed alignment). (TIF)

S7 Fig Clade-specific presentation of the sequence logos for the DAF-12 ligand binding domain (LBD) (positions 1897–2198 of the untrimmed alignment). (TIF)

S1 Table Thermal stabilization of the *Brugia malayi* (*Bma*) and *Haemonchus contortus* (*Hco*) DAF-12 ligand-binding domains (LBDs) following ligand incubation. Inflection temperatures deduced from nano-DSF experiments (Figs 1B, S2A and S2B) of the *Bma*DAF-12 and *Hco*DAF-12 LBDs in their apo state and in the presence of one and three molar excess of ∆4-DA and ∆7-DA.
(TIF)

S2 Table X-ray data collection and refinement statistics for *Brugia malayi* (*Bma*) and *Haemonchus contortus* (*Hco*) DAF-12. Data were collected from one crystal for each dataset. Values in parentheses are for the highest-resolution shell.
(DOCX)

S3 Table Ligand binding pocket volumes (Å3) of the ligand-binding domains (LBDs) of human hLXR, hFXR, and hVDR, and of *Brugia malayi* (*Bma*) DAF-12, *Haemonchus contortus* (*Hco*) DAF-12, *Ancylostoma ceylanicum* (*Ace*) DAF-12, and *Strongyloides stercoralis* (*Sst*) DAF-12. Volumes were estimated using KVFinder-web.
(DOCX)

S4 Table Conservation of hydrophobic amino acids involved in ligand binding in DAF-12 from *Brugia malayi* (*Bma*), *Haemonchus contortus* (*Hco*), *Strongyloides stercoralis* (*Sst*), and *Ancylostoma ceylanicum* (*Ace*). The last column gives the correspondence with the sequence alignment numbering in Fig 4.
(TIF)

S5 Table Sequences and accession numbers of the DAF-12 proteins used in this analysis. (DOCX)

S1 File Sequence alignment of complete DAF-12 proteins. (FASTA)

S2 File Trimmed sequence alignment of DAF-12 proteins used to generate the phylogenetic tree. (FASTA)

S1 Raw Gel Original images of SDS-PAGE gels corresponding to the size exclusion chromatography purification step, used to generate S1A and S1B Fig. (PDF)
