# Supplementary material for: Structural and evolutionary insights into DAF-12 interactions with transcriptional coactivators in parasitic nematodes
Source: PLoS Pathog. 2026 Jul 20;22(7):e1014073. doi: 10.1371/journal.ppat.1014073 (PMC13568512; doi:10.1371/journal.ppat.1014073)
Supplement: Supplementary file 1 [file ppat.1014073.s015.pdf]

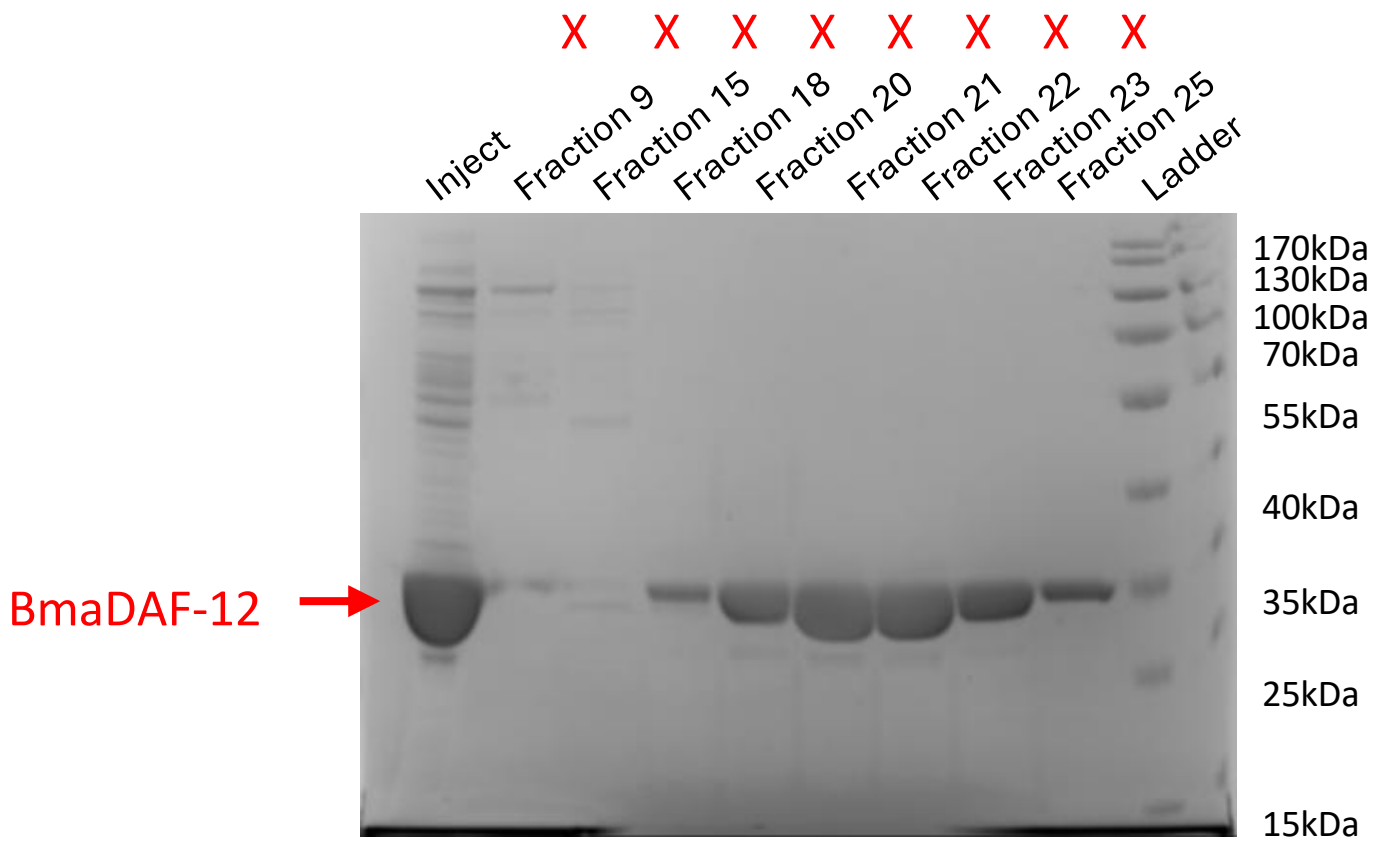

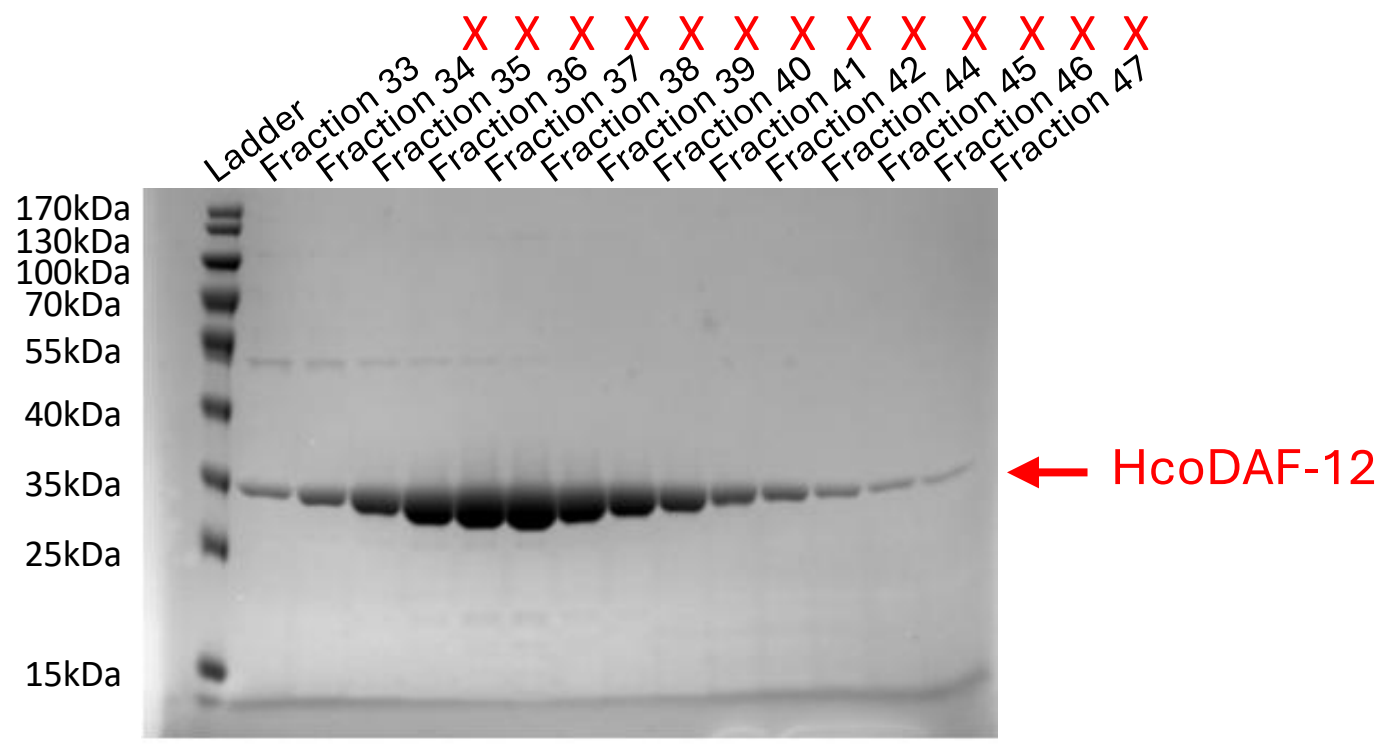

SDS-PAGE imaged using a Gel Doc EZ™ Imager from BIO-RAD and the White Sample Tray.
The gels were scanned using ImageLab 5.1 software and the Comassie Blue protocol.
SDS-PAGE correpond to the size exclusion chromatrography purification step.
Original images used to generated Supplementary figures S1A and S1B.
