# Supplementary material for: Structural and evolutionary insights into DAF-12 interactions with transcriptional coactivators in parasitic nematodes
Source: PLoS Pathog. 2026 Jul 20;22(7):e1014073. doi: 10.1371/journal.ppat.1014073 (PMC13568512; doi:10.1371/journal.ppat.1014073)
Supplement: Supplementary file 2 [file ppat.1014073.s009.docx]

|  | ***Bma*DAF-12**  **Δ4-dafachronic acid - PGC1α-1** | ***Hco*DAF-12**  **Δ4-dafachronic acid – SRC2-2** |
| --- | --- | --- |
| **Pdb code** | 9TLX | 9TL4 |
| **Data collection** |  |  |
| **Beamline** | ID-30B | ID-30B |
| **Wavelength (Å)** | 0.969 | 0.969 |
| **Space group** | P2_1_2_1_2_1_ | P2_1_2_1_2_1_ |
| **Cell dimensions** |  |  |
| **a, b, c (Å)** | 77.29, 77.75, 90.20 | 39.52, 74.23, 92.21 |
| **α, β, γ (°)** | 90, 90, 90 | 90, 90, 90 |
| **Resolution range (Å)** | 45.10 – 1.70 (1.76 – 1.7) | 39.52 – 2.0 (2.07 – 2.0) |
| **Rsym** | 0.0614 (0.365) | 0.0465 (0.4942) |
| **I / αI** | 10.71 (2.43) | 14.49 (1.70) |
| **Completeness (%)** | 99.88 (99.83) | 90.87 (59.55) |
| **Redundancy** | 2.0 (2.0) | 1.9 (1.6) |
| **Refinement** |  |  |
| **Resolution range (Å)** | 45.10 – 1.70 (1.76 – 1.7) | 39.52 – 2.0 (2.07 – 2.0) |
| **No. reflections** | 60374 (5948) | 33162 (1816) |
| **R_work_ / R_free_** | 17.54% / 21.01% | 17.82% / 21.28% |
| **No. atoms** | 4941 | 2257 |
| **Protein** | 4156 | 2023 |
| **Ligand/ion** | 142 | 44 |
| **Water** | 643 | 190 |
| **B-factor** | 20.74 | 29.02 |
| **Protein** | 19.28 | 28.36 |
| **Ligand/ion** | 18.97 | 27.75 |
| **Water** | 30.55 | 36.26 |
| **Ramachandran favored (%)** | 99.01 | 98.80 |
| **Ramachandran allowed (%)** | 0.99 | 1.20 |
| **Ramachandran outliers (%)** | 0.00 | 0.00 |
| **R.m.s. deviations** |  |  |
| **Bond length (Å)** | 0.007 | 0.012 |
| **Bond angles (°)** | 1.12 | 1.23 |
