# Supplementary material for: Structural and evolutionary insights into DAF-12 interactions with transcriptional coactivators in parasitic nematodes
Source: PLoS Pathog. 2026 Jul 20;22(7):e1014073. doi: 10.1371/journal.ppat.1014073 (PMC13568512; doi:10.1371/journal.ppat.1014073)
Supplement: Supplementary file 3 [file ppat.1014073.s010.docx]

|  | hLXR (3kfc) | hFXR (3l1b) | hVDR (1db1) | *Bma*DAF-12 (9tlx) | *Hco*DAF-12 (9tl4) | *Ace*DAF-12 (3up3) | *Ace*DAF-12 (3up0) | *Sst*DAF-12 (3gyt) | *Sst*DAF-12 (3gyu) |
| --- | --- | --- | --- | --- | --- | --- | --- | --- | --- |
| V (Å^3^) | 569/599/616 | 461 | 551 | 718/724 | 666 | 767 | 699/676 | 655 | 762 |
