# Supplementary material for: Structural and evolutionary insights into DAF-12 interactions with transcriptional coactivators in parasitic nematodes
Source: PLoS Pathog. 2026 Jul 20;22(7):e1014073. doi: 10.1371/journal.ppat.1014073 (PMC13568512; doi:10.1371/journal.ppat.1014073)
Supplement: Supplementary file 4 [file ppat.1014073.s012.docx]

| **Name** | **Species name** | **Clade** | **Accession** |
| --- | --- | --- | --- |
| A. besseyi_PRJNA834627\|M3Y9400792600 | Aphelenchoides besseyi (PRJNA834627 - APVT) | IV | M3Y94_00792600 |
| A. bicaudatus_PRJNA834627\|M3Y97 00254200 | Aphelenchoides bicaudatus (PRJNA834627 - Fsh) | IV | M3Y97_00254200 |
| A. caninum_PRJNA72585\|ANCCAN15412 | Ancylostoma caninum (PRJNA72585 - Baltimore) | V | ANCCAN_15412 |
| A. ceylanicum_PRJNA231479\|Aceys0032v2g6728 | Ancylostoma ceylanicum (PRJNA231479 - HY135) | V | Acey_s0032.v2.g6728 |
| A. costaricensis_PRJEB494\|ACOC 0000064201 | Angiostrongylus costaricensis (PRJEB494 - Costa Rica) | V | ACOC_0000064201 |
| A. duodenale_PRJNA72581\|ANCDUO17434 | Ancylostoma duodenale (PRJNA72581 - Baltimore) | V | ANCDUO_17434 |
| A. fujianensis_PRJNA834627\|M3Y9900070200 | Aphelenchoides fujianensis (PRJNA834627 - Dali) | IV | M3Y99_00070200 |
| A. lumbricoides_PRJEB4950\|ALUE0001871801-ALUE0001821701 | Ascaris lumbricoides (PRJEB4950 - Republic of Ecuador) | III | ALUE_0001871801 & ALUE_0001821701 |
| A. nanus_PRJEB26554\|ACRNANscaffold3570 g20078 | Acrobeloides nanus (PRJEB26554) | IV | ACRNAN_scaffold3570.g20078 |
| A. simplex_PRJEB496\|ASIM0001146101 | Anisakis simplex (PRJEB496) | III | ASIM_0001146101 |
| A. sudhausi_PRJEB48369_1\|ALDISUDHAUS000001025 | Allodiplogaster sudhausi (PRJEB48369 - SB413) | V | ALDISUDHAUS000001025 |
| A. sudhausi_PRJEB48369_2\|ALDISUDHAUS000001054 | Allodiplogaster sudhausi (PRJEB48369 - SB413) | V | ALDISUDHAUS000001054 |
| A. suum_PRJNA62057\|AgR054g014 | Ascaris suum (PRJNA62057 - AG01) | III | AgR054_g014 |
| A. vasorum_PRJNA663250\|AngVas-contig57-13 | Angiostrongylus vasorum (PRJNA663250 - ZH-2019-22) | V | AngVas-contig_57-1.3 |
| A. viteae_PRJEB1697\|nAv101g08097 | Acanthocheilonema viteae (PRJEB1697) | III | nAv.1.0.1.g08097 |
| AuanemaJU1783_PRJEB51845\|JU1783 LOCUS10797 | Auanema sp. JU1783 (PRJEB51845 - JU1783) | V | JU1783_LOCUS10797 |
| B. listronoti_PRJNA842945\|Bralisg11736 | Bradynema listronoti (PRJNA842945 - AAFC-BrLi-01_J3_LR_R9) | V | Bralis.g11736 |
| B. malayi_PRJNA10729\|Bm8452WBGene00228713 | Brugia malayi (PRJNA10729 - FR3) | III | Bm8452_WBGene00228713 |
| B. okinawaensis_PRJEB40023\|BOKI060136000 | Bursaphelenchus okinawaensis (PRJEB40023 - SH1) | IV | BOKI.060136000 |
| B. xylophilus_PRJEA64437\|BXY1418300 | Bursaphelenchus xylophilus (PRJEA64437) | IV | BXY_1418300 |
| B. xylophilus_PRJEB40022\|BXYJ5060111900 | Bursaphelenchus xylophilus (PRJEB40022 - Ka4C1) | IV | BXYJ5.060111900 |
| C. angaria_PRJNA51225\|Cang2012031300091g4057 | Caenorhabditis angaria (PRJNA51225) | V | Cang_2012_03_13_00091.g4057 |
| C. auriculariae_PRJEB40642\|CAUJLOCUS4595 | Caenorhabditis auriculariae (PRJEB40642 - NKZ352) | V | CAUJ_LOCUS4595 |
| C. becei_PRJEB28243\|CSP29g15982 | Caenorhabditis becei (PRJEB28243 - QG2083) | V | CSP29.g15982 |
| C. bovis_PRJEB34497\|CBOVIg3971 | Caenorhabditis bovis (PRJEB34497) | V | CBOVI.g3971 |
| C. brenneri_PRJNA20035\|CBN31767WBGene00158906 | Caenorhabditis brenneri (PRJNA20035) | V | CBN31767_WBGene00158906 |
| C. briggsae_PRJNA10731\|CBG04512aWBGene00027166 | Caenorhabditis briggsae (PRJNA10731) | V | CBG04512a_WBGene00027166 |
| C. elegans_PRJNA13758\|F11A13aWBGene00000908 | Caenorhabditis elegans (PRJNA13758 - N2) | V | F11A1.3a_WBGene00000908 |
| C. inopinata_PRJDB5687\|Sp34X0074510 | Caenorhabditis inopinata (PRJDB5687) | V | Sp34_X0074510 |
| C. japonica_PRJNA12591\|CJA11532WBGene00130736 | Caenorhabditis japonica (PRJNA12591) | V | CJA11532_WBGene00130736 |
| C. johnstoni_PRJEB47283\|CJOHNSTONILOCUS2016 | Cercopithifilaria johnstoni (PRJEB47283) | III | CJOHNSTONI_LOCUS2016 |
| C. latens_PRJNA248912\|FL83 01282 | Caenorhabditis latens (PRJNA248912) | V | FL83_01282 |
| C. nassatus_PRJEB63274\|CYNASLOCUS21369 | Cylicocyclus nassatus (PRJEB63274 - Nouzilly) | V | CYNAS_LOCUS21369 |
| C. nigoni_PRJNA384657\|Cni-daf-12 | Caenorhabditis nigoni (PRJNA384657 - JU1422) | V | Cni-daf-12 |
| C. panamensis_PRJEB28259\|CSP28g5501 | Caenorhabditis panamensis (PRJEB28259) | V | CSP28.g5501 |
| C. parvicauda_PRJEB12595\|CSP21g10182 | Caenorhabditis parvicauda (PRJEB12595) | V | CSP21.g10182 |
| C. quiockensis_PRJEB11354\|CSP38g8805 | Caenorhabditis quiockensis (PRJEB11354 - JU2809) | V | CSP38.g8805 |
| C. remanei_PRJNA53967\|CRE07237WBGene00058429 | Caenorhabditis remanei (PRJNA53967) | V | CRE07237_WBGene00058429 |
| C. remanei_PRJNA577507\|GCK72 023616 | Caenorhabditis remanei (PRJNA577507 - PX506) | V | GCK72_023616 |
| C. sinica_PRJNA194557\|Csp5 scaffold00063g3034 | Caenorhabditis sinica (PRJNA194557) | V | Csp5_scaffold_00063.g3034 |
| C. sulstoni_PRJEB12601\|CSP32g2065 | Caenorhabditis sulstoni (PRJEB12601 - JU2788) | V | CSP32.g2065 |
| C. tribulationis_PRJEB12608\|CSP40g20318 | Caenorhabditis tribulationis (PRJEB12608 - JU2818) | V | CSP40.g20318 |
| C. tropicalis_PRJNA53597\|Csp11Scaffold629g13785 | Caenorhabditis tropicalis (PRJNA53597) | V | Csp11.Scaffold629.g13785 |
| C. uteleia_PRJEB12600\|CSP31g25010 | Caenorhabditis uteleia (PRJEB12600 - JU2585) | V | CSP31.g25010 |
| C. waitukubuli_PRJEB12602\|CSP39g8512 | Caenorhabditis waitukubuli (PRJEB12602 - NIC564) | V | CSP39.g8512 |
| C. zanzibari_PRJEB12596\|CSP26g3116 | Caenorhabditis zanzibari (PRJEB12596 - JU2190) | V | CSP26.g3116 |
| D. coronatus_PRJDB31431\|DCO004589 | Diploscapter coronatus (PRJDB3143 - PDL0010) | V | DCO_004589 |
| D. destructor_PRJNA312427\|Dd05346 | Ditylenchus destructor (PRJNA312427 - Dd01) | IV | Dd_05346 |
| D. immitis_PRJEB1797\|nDi222g09652 | Dirofilaria immitis (PRJEB1797) | III | nDi.2.2.2.g09652 |
| D. magnus_PRJNA655932\|DMAGNUSscaf00050g000997 | Diplogasteroides magnus (PRJNA655932 - TMG144) | V | DMAGNUSscaf00050g000997 |
| D. medinensis_PRJEB500\|DME0000943901 | Dracunculus medinensis (PRJEB500) | III | DME_0000943901 |
| D. pachys_PRJNA280107\|WR2516540 | Diploscapter pachys (PRJNA280107 - PF1309) | V | WR25_16540 |
| D. viviparus_PRJNA72587\|DICVIV07098 | Dictyocaulus viviparus (PRJNA72587) | V | DICVIV_07098 |
| E. elaphi_PRJEB502\|EEL0000446201 | Elaeophora elaphi (PRJEB502) | III | EEL_0000446201 |
| E. vermicularis_PRJEB503\|EVEC0001257601 | Enterobius vermicularis (PRJEB503 - Spain/Canary Islands) | III | EVEC_0001257601 |
| G. rostochiensis_PRJNA695196\|Gr19v10g11452 | Globodera rostochiensis (L19 PRJNA695196) | IV | Gr19_v10_g11452 |
| H. contortus_PRJEB506\|HCON00164850 | Haemonchus contortus (PRJEB506) | V | HCON_00164850 |
| H. glycines_PRJNA381081\|Hetgly12168 | Heterodera glycines (PRJNA381081 - TN10) | IV | Hetgly12168 |
| H. mephisto_PRJNA528747\|MSTRG10611 | Halicephalobus mephisto (PRJNA528747 - m2B) | IV | MSTRG.10611 |
| H. polygyrus_PRJEB15396\|HPOL0001414101 | Heligmosomoides polygyrus (PRJEB15396) | V | HPOL_0001414101 |
| H. schachtii_PRJNA522950\|Hscgene16109 | Heterodera schachtii (PRJNA522950 - Bonn) | IV | Hsc_gene_16109 |
| HalicephalobusNKZ332_PRJNA555616\|FO519006023 | Halicephalobus sp. NKZ332 (PRJNA555616 - NKZ332) | IV | FO519_006023 |
| K. luziae_PRJNA655932\|KOLUg446 | Koerneria luziae (PRJNA655932 - NKZ323) | V | KOLU_g446 |
| L. loa_PRJNA246086\|EN7012190 | Loa loa (PRJNA246086 - CAT) | III | EN70_12190 |
| L. sigmodontis_PRJEB3075\|nLs212g07694 | Litomosoides sigmodontis (PRJEB3075 - Bain lab strain) | III | nLs.2.1.2.g07694 |
| L. texanum_PRJNA655932\|LTEXAscaf05760g019681 | Levipalatum texanum (PRJNA655932 - TMG5) | V | LTEXAscaf05760g019681 |
| M. arenaria_PRJNA438575_1\|tig00001238g15364 | Meloidogyne arenaria (PRJNA438575 - A2-O Okinawa_01) | IV | tig00001238.g15364 |
| M. arenaria_PRJNA438575_2\|tig00001693g48918 | Meloidogyne arenaria (PRJNA438575 - A2-O Okinawa_01) | IV | tig00001693.g48918 |
| M. belari_PRJEB61636\|MBELARILOCUS7344 | Mesorhabditis belari (PRJEB61636 - JU2817) | V | MBELARI_LOCUS7344 |
| M. enterolobii_PRJEB36431_1\|MENTLOCUS16462 | Meloidogyne enterolobii (PRJEB36431) | IV | MENT_LOCUS16462 |
| M. enterolobii_PRJEB36431_2\|MENTLOCUS24022 | Meloidogyne enterolobii (PRJEB36431) | IV | MENT_LOCUS24022 |
| M. enterolobii_PRJEB36431_3\|MENTLOCUS40684 | Meloidogyne enterolobii (PRJEB36431) | IV | MENT_LOCUS40684 |
| M. floridensis_PRJNA340324\|scf7180000423856g11745 | Meloidogyne floridensis (PRJNA340324 - SJF1) | IV | scf7180000423856.g11745 |
| M. graminicola_PRJNA411966\|NXFT0100033418827g | Meloidogyne graminicola (PRJNA411966) | IV | NXFT01000334.1.8827_g |
| M. incognita_PRJEB8714_1\|Minc3s00350g10770 | Meloidogyne incognita (PRJEB8714) | IV | Minc3s00350g10770 |
| M. incognita_PRJEB8714_2\|Minc3s01435g23791 | Meloidogyne incognita (PRJEB8714) | IV | Minc3s01435g23791 |
| M. japonica_PRJEB27334\|MĲAPON000001950 | Micoletzkya japonica (PRJEB27334) | V | MIJAPON000001950 |
| M. javanica_PRJNA340324_1\|scaffold34667cov245g21526 | Meloidogyne javanica (PRJNA340324 - VW4) | IV | scaffold34667_cov245.g21526 |
| M. javanica_PRJNA340324_2\|scaffold4683cov250g8542 | Meloidogyne javanica (PRJNA340324 - VW4) | IV | scaffold4683_cov250.g8542 |
| M. spiculigera_PRJEB59059_1\|MSPICULIGERALOCUS1110 | Mesorhabditis spiculigera (PRJEB59059 - AF72) | V | MSPICULIGERA_LOCUS1110 |
| M. spiculigera_PRJEB59059_2\|MSPICULIGERALOCUS12305 | Mesorhabditis spiculigera (PRJEB59059 - AF72) | V | MSPICULIGERA_LOCUS12305 |
| N. americanus_PRJNA1007425\|NecatorchrX pre1g21353 | Necator americanus (PRJNA1007425 - Aroian) | V | Necator_chrX.pre1.g21353 |
| N. brasiliensis_PRJNA994163\|NippochrX g15301 | Nippostrongylus brasiliensis (PRJNA994163 - MIMR) | V | Nippo_chrX.g15301 |
| O. tipulae_PRJEB15512\|OTIPUnOt201g01766 | Oscheius tipulae (PRJEB15512) | V | OTIPU.nOt.2.0.1.g01766 |
| O. tipulae_PRJNA644888\|Oti16230g | Oscheius tipulae (PRJNA644888 - CEW1) | V | Oti_16230_g |
| O. volvulus_PRJEB513\|OVOC2228 | Onchocerca volvulus (PRJEB513 - O. volvulus Cameroon isolate) | III | OVOC2228_WBGene00239037 |
| P. arcanus_PRJEB27334\|PARCANUS000000445 | Pristionchus arcanus (PRJEB27334) | V | PARCANUS000000445 |
| P. entomophagus_PRJEB273341\|PRENTOPHAG000001498 | Pristionchus entomophagus (PRJEB27334) | V | PRENTOPHAG000001498 |
| P. exspectatus_PRJEB46690\|PREXSPEC000000910 | Pristionchus exspectatus (PRJEB46690) | V | PREXSPEC000000910 |
| P. fissidentatus_PRJEB27334\|PRIFISSI000004826 | Pristionchus fissidentatus (PRJEB27334) | V | PRIFISSI000004826 |
| P. japonicus_PRJEB27334\|TRINITYc14910g1 i4loc1 | Pristionchus japonicus (PRJEB27334) | V | TRINITY_c14910_g1_i4_loc1 |
| P. maxplancki_PRJEB27334\|PRIMPAX000000819 | Pristionchus maxplancki (PRJEB27334) | V | PRIMPAX000000819 |
| P. mayeri_PRJEB27334\|PRIMAYER000000843 | Pristionchus mayeri (PRJEB27334) | V | PRIMAYER000000843 |
| P. pacificus_PRJNA12644\|PPA28648WBGene00118202 | Pristionchus pacificus (PRJNA12644 - PS312) | V | PPA28648_WBGene00118202 |
| P. redivivus_PRJNA186477\|Pang1544 | Panagrellus redivivus (PRJNA186477) | IV | Pan_g1544 |
| P. sambesii_PRJNA390260\|PSAMBscaffold462size50287g6186 | Plectus sambesii (PRJNA390260 - ES601) | C | PSAMB.scaffold462size50287.g6186 |
| P. superbus_PRJEB32708\|PSUv2g12710 | Panagrolaimus superbus (PRJEB32708) | IV | PSU_v2.g12710 |
| P. trichosuri_PRJEB515\|PTRK0001378700 | Parastrongyloides trichosuri (PRJEB515 - KNP) | IV | PTRK_0001378700 |
| P. univalens_PRJNA386823\|PgB19g025 | Parascaris univalens (PRJNA386823 - PG01) | III | PgB19_g025 |
| Panagrolaimus1159_PRJEB32708\|PS1159v2g19594 | Panagrolaimus sp. 1159 (PRJEB32708) | IV | PS1159_v2.g19594 |
| Rhabditophanes_KR3021PRJEB1297\|RSKR0001015700 | Rhabditophanes sp. KR3021 (PRJEB1297 - kr 3021) | IV | RSKR_0001015700 |
| S. digitata_PRJNA479729\|sdigicontig832g9863 | Setaria digitata (PRJNA479729 - RSCHEM2018) | III | sdigi.contig832.g9863 |
| S. feltiae_PRJNA3536101\|contig00125025 | Steinernema feltiae (PRJNA353610 - NW) | IV | contig00125.0.25 |
| S. glaseri_PRJNA204943\|L893g20420 | Steinernema glaseri (PRJNA204943) | IV | L893_g20420 |
| S. hermaphroditum_PRJNA982879_1\|Sherm Ng9231 | Steinernema hermaphroditum (PRJNA982879 - PS9179) | IV | Sherm_N.g9231 |
| S. hermaphroditum_PRJNA982879_2\|Sherm Xg16888 | Steinernema hermaphroditum (PRJNA982879 - PS9179) | IV | Sherm_X.g16888 |
| S. monticolum_PRJNA205067_1\|L898g10498 | Steinernema monticolum (PRJNA205067) | IV | L898_g10498 |
| S. monticolum_PRJNA205067_2\|L898g9012 | Steinernema monticolum (PRJNA205067) | IV | L898_g9012 |
| S. muris_PRJEB524\|SMUV0001002101 | Syphacia muris (PRJEB524 - Spain/Valencia) | III | SMUV_0001002101 |
| S. papillosus_PRJEB525\|SPAL0001591300 | Strongyloides papillosus (PRJEB525 - LIN) | IV | SPAL_0001591300 |
| S. ratti_PRJEB125\|SRAE 0000032100 | Strongyloides ratti (PRJEB125) | IV | SRAE_0000032100_WBGene00256064 |
| S. scapterisci_PRJNA204942_1\|L892g18386 | Steinernema scapterisci (PRJNA204942) | IV | L892_g18386 |
| S. scapterisci_PRJNA204942_2\|L892g20774 | Steinernema scapterisci (PRJNA204942) | IV | L892_g20774 |
| S. stercoralis_PRJNA930454\|XLOC002244 | Strongyloides stercoralis (PRJNA930454 - PV001) | IV | XLOC_002244 |
| S. venezuelensis_PRJEB530\|SVE 0996600 | Strongyloides venezuelensis (PRJEB530) | IV | SVE_0996600 |
| T. canis_PRJNA248777\|Tcan08729 | Toxocara canis (PRJNA248777 - PN_DK_2014) | III | Tcan_08729 |
| T. circumcincta_PRJNA72569\|TELCIR05516 | Teladorsagia circumcincta (PRJNA72569) | V | TELCIR_05516 |
| W. bancrofti_PRJNA275548\|Wban03033 | Wuchereria bancrofti (PRJNA275548 - 17-3) | III | Wban_03033 |
